# Designing Tissue Function Through Embedded Bioprinting Architectures

**DOI:** 10.1002/advs.77342

**Published:** 2026-08-25

**Authors:** Qi Li, Yifei Cao, Jiaxin Lin, Huayong Yang, Hongzhao Zhou

**Affiliations:** ^1^ School of Engineering Hangzhou Normal University Hangzhou People's Republic of China; ^2^ State Key Laboratory of Fluid Power and Mechatronic Systems Zhejiang University Hangzhou People's Republic of China; ^3^ School of Mechanical Engineering Zhejiang University Hangzhou People's Republic of China; ^4^ State Key Laboratory of Materials Processing and Die & Mould Technology Huazhong University of Science and Technology Wuhan People's Republic of China

**Keywords:** 3D bioprinting, biofabrication, embedded bioprinting, tissue engineering, tissue models

## Abstract

Embedded Bioprinting (EB) enables the fabrication of soft, cell‐dense tissues within mechanically supportive environments, substantially expanding the design space of biofabrication. While EB has rapidly emerged as a powerful strategy for printing structures that are otherwise unattainable by freeform approaches, current progress is often evaluated in terms of geometric complexity rather than functional relevance. As the field evolves, a central challenge is not simply whether complex architectures can be printed, but how printing‐defined structures can be intentionally designed to support tissue‐specific functions. In contrast to previous reviews that primarily organize EB by supporting bath formulations, material systems, or printing modalities, this review adopts a function‐guided design framework that organizes EB according to the architectural features required to support tissue‐specific function. Within this framework, target tissue functions are first decomposed into structural and microarchitectural requirements, which then define fabrication objectives and guide the selection of supporting bath mechanics, material formulations, crosslinking strategies, and process parameters. Across diverse tissue systems, this framework reveals emerging structure–process–function relationships and key limitations in predictability and standardization, providing a conceptual basis for reproducible, human‐relevant disease models and drug evaluation platforms while informing longer‐term regenerative and implantable applications.

## Graphical abstract

Embedded bioprinting enables soft, cell‐dense constructs to be fabricated within supporting bath environments. This review introduces a function‐guided framework linking target tissue functions to architectural requirements, supporting bath selection, bioink design, printing dynamics, and validation assays. By comparing cardiac, skeletal muscle, bone and cartilage, vascular, epithelial and parenchymal organs, it highlights how printed architectures may support maturation, physiological function, standardization, and future human‐relevant tissue modeling.

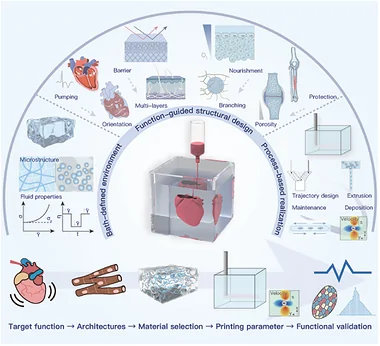

## 1 Introduction

Living tissues derive function from architecture. Cellular organization, interfacial continuity, transport pathways, and spatial heterogeneity collectively establish the basic conditions that enable physiological behavior. For instance, aligned cardiomyocytes within anisotropic architectures enable coordinated contraction [1]. Layered, polarized epithelia support selective exchange and protection [2]. Hierarchical vascular networks sustain metabolism and coordinate tissue‐scale function [3, 4]. In bone, gradients in pore size, mineralization, and stiffness create heterogeneous mechanical environments that support load bearing [5, 6]. These architectural features constrain how tissue‐specific function subsequently emerges.

**FIGURE 1:**
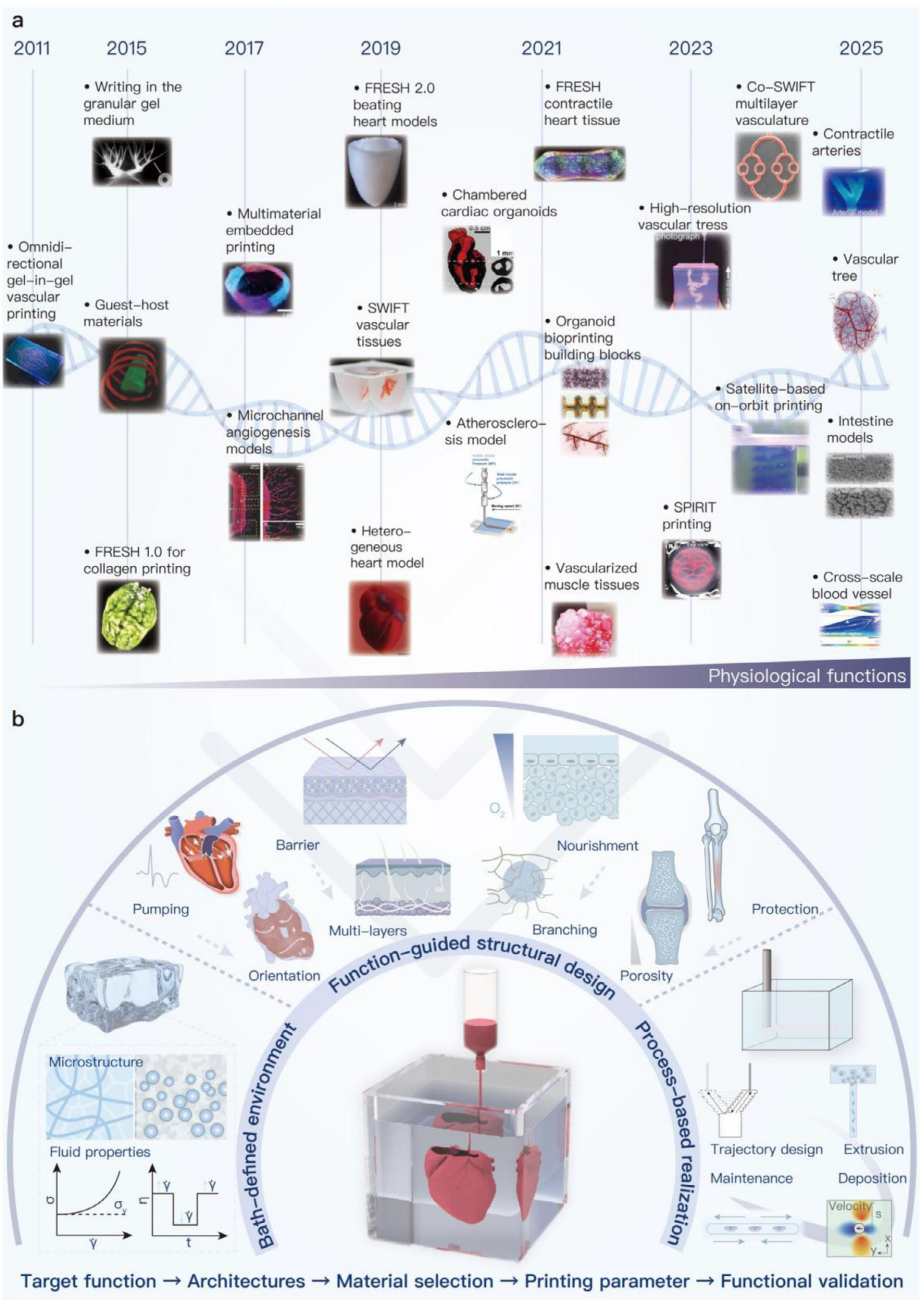
Function‐guided evolution of embedded bioprinting. (a) The historical progression of embedded bioprinting (2011–2025). (b) A function‐guided design framework that links target tissue function, required architecture, supporting bath and bioink selection, printing parameters, and tissue‐specific validation assays. Elements in (a) were reproduced with permission from [2], Copyright 2025, The American Association for the Advancement of Science [9], Copyright 2019, The American Association for the Advancement of Science [10], Copyright 2011, John Wiley and Sons [12], Copyright 2019, The American Association for the Advancement of Science [14], Copyright 2015, John Wiley and Sons [15], Copyright 2015, The American Association for the Advancement of Science [16], Copyright 2018, John Wiley and Sons [18], Copyright 2020, John Wiley and Sons [19], Copyright 2020, Wolters Kluwer Health, LLC [20], Copyright 2020, Springer Nature [22], Copyright 2022, IOP Publishing Limited [23], Copyright 2023, The American Association for the Advancement of Science [24], Copyright 2023, John Wiley and Sons [25], Copyright 2024, John Wiley and Sons [26], Copyright 2025, Elsevier [28], Copyright 2025, The American Association for the Advancement of Science [29], Copyright 2018, Elsevier and [30], Copyright 2023, John Wiley and Sons. Elements from [13], [17], and [21] were reproduced or adapted under the terms of the Creative Commons Attribution license, Copyright 2015, The Authors Copyright 2019, The Authors, published by Wiley‐VCH Verlag GmbH & Co. KGaA; and Copyright 2021, The Author(s), published by Springer Nature, respectively. The element from [27] is reproduced from the authors' own previously published work. Copyright 2025, The Authors, published by Elsevier Inc.

EB has expanded the ability of conventional three‐dimensional (3D) bioprinting [7, 8] to fabricate such architectures by enabling soft, cell‐dense bioinks to be deposited within mechanically supportive environments [9]. Early work established that complex geometries could be fabricated without requiring inks to be self‐supporting, positioning EB as a powerful approach for freeform tissue construction [10, 11, 12]. As supporting baths and printing processes have become more controllable, the feasibility of printing complex structures has largely been established (Figure 1a). The EB here is defined as printing strategies in which a supporting bath stabilizes deposited inks during or immediately after freeform deposition. This scope includes granular, continuum, and all‐aqueous supporting baths, as well as sacrificial, coaxial, or hybrid strategies when the supporting bath is essential for preserving freeform architecture, lumen continuity, or multilayered organization. Purely extrusion‐based or volumetric printing strategies without embedded deposition are outside the main scope of this review.

As EB has matured, the central challenge has shifted from whether structures can be printed to how architectural features established during printing can be designed to support tissue‐specific function. Most reviews continue to organize the field around supporting bath formulations or printing performance. While these perspectives clarify key technical principles, they often stop short of linking printing strategies to the structural requirements of specific tissues, leaving the relationship between fabrication decisions and functional outcomes implicit and limiting the development of functional tissue fabrication.

Here, we organize EB around a function‐guided design framework (Figure 1b) that connects tissue‐level objectives to fabrication decisions. In this framework, target tissue function first defines the architectural features that must be established, such as cellular organization, interface continuity, transport hierarchy, spatial heterogeneity, lumen continuity, or perfusable pathways. These architectural requirements are then translated into fabrication targets that guide the selection of supporting bath mechanics, bioink formulations, crosslinking strategies, and printing parameters. The resulting constructs should be evaluated using tissue‐specific validation assays, including contraction, conduction, permeability, vasoreactivity, metabolic activity, transport function, or drug response depending on the intended tissue model. By applying this workflow across cardiac, skeletal muscle, bone and cartilage, vascular, epithelial, tubular, and parenchymal tissues, this review examines how microarchitectural control supports tissue‐level function and identifies key gaps that limit reproducible, human‐relevant EB platforms.

## 2 Supporting Baths as Formation Environments

**FIGURE 2:**
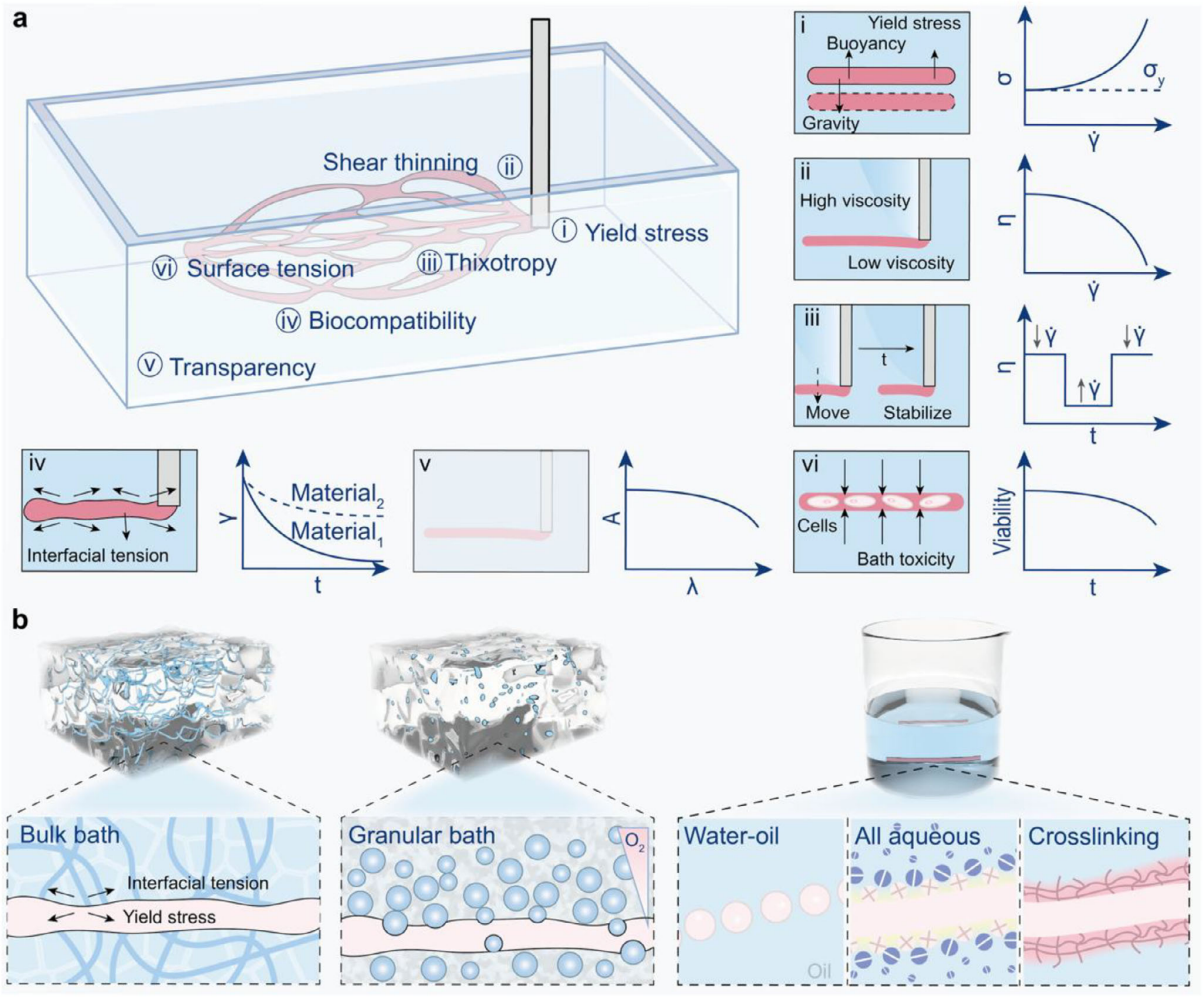
Supporting baths as formation environments. (a) Key properties of the supporting bath and their influence on printing. (b) Classification of existing supporting baths.

Within a function‐guided framework, supporting baths are selected according to the structural features a tissue requires, which determine whether physiological function can be achieved. The following discussion focuses on how bath mechanics constrain the types of tissue‐relevant structures that can be reliably encoded (Figure 2).

### 2.1 Supporting Bath Properties and Their Influences on Printing Behavior

#### 2.1.1 Yield Stress

Yield stress determines the ability of a supporting bath to stabilize deposited bioinks and preserve continuous, load‐bearing, or perfusable structures required for tissue function. It influences bioink support [31, 32], nozzle‐induced disturbance [15], deposited filament shape [33, 34], and coalescence between adjacent filaments [33]. Experimentally, yield stress is obtained by fitting steady‐shear data to the Herschel–Bulkley model (H–B, *σ =* *σ*_y _+ *kγ̇^n^*, where *σ* is the shear stress, *σ*_y_ is the yield stress, *k* is the flow consistency index, *n* is the flow behavior index, and *γ̇* is the shear rate) [15] or by locating the intersection of storage and loss moduli in oscillatory measurements [35]. Although these methods may report slightly different numerical values [36], they capture the same transition from elastic to plastic behavior.

Baths with insufficient yield stress fail to support vertical features, whereas excessively high yield stress limits crevasse recovery. Reported EB systems commonly use supporting baths with yield stress values on the order of 1–100 Pa, often lower than those of the deposited bioinks [15, 37]. However, the optimal range is system‐dependent and varies with bioink viscosity, nozzle diameter, printing speed, immersion depth, crosslinking kinetics, and the target architecture. Within this range, filaments could maintain round cross sections, merge predictably, and produce continuous features that underlie perfusable channels and anisotropic mechanical function.

#### 2.1.2 Shear Thinning

Shear thinning permits the bath to fluidize locally under applied shear during nozzle motion, while maintaining mechanical stability in the absence of stress [38]. This arises from particle rearrangements or polymer disentanglement [35], captured by the flow behavior index *n* in the H–B model. The *n* value has been reported to follow a linear relationship with lg(*σ*_y_) [39]. Lower *n* indicates stronger shear‐thinning, and reported EB supporting baths often show *n* values in the approximate range of 0.1 ≤ *n *≤ 0.9 [40, 41, 42, 43, 44].

Pronounced shear thinning lowers the lateral resistance on the nozzle, which prevents nozzle bending and improves spatial positioning precision. The associated decrease in extrusion pressure promotes smooth flow and supports uniform filament diameters [45]. After nozzle passage, viscoelastic recovery of the bath stabilizes the deposited filament and preserves geometry. These combined effects establish the structural accuracy needed for later functional development, such as synchronized contraction or gradient mechanical distribution.

#### 2.1.3 Thixotropy

Thixotropy describes the rate at which structure is rebuilt after shear‐induced breakdown. It determines the temporal stability of printed architecture by controlling how rapidly the bath recovers after shear and stabilizes newly deposited filaments. Recovery times are typically measured through three‐step rheological tests (low‐high‐low shear rate [46] or strain [47] with 50%–100% recovery threshold [33, 48, 49, 50, 51]). In several reported systems, recovery times on the order of 0.2–1 s have been used to balance rapid architectural preservation and avoidance of trapped voids [33, 47, 52]. The preferred recovery time depends on nozzle speed, filament diameter, bath depth, ink viscosity, and whether the target structure requires discrete filaments, fused walls, or smooth lumens.

Both shear thinning and thixotropic recovery define how the bath transiently yields during nozzle motion and how rapidly it stabilizes printed features afterward. This temporal control determines whether architecture is preserved across repeated trajectories, directly affecting feature fidelity and spatial organization.

#### 2.1.4 Surface Tension

Surface tension (*γ*) at the interface between the bath and bioink regulates filament smoothness and continuity, shaping interface quality that is critical for transport and barrier‐related tissue functions. Its interaction with yield stress determines the smallest stable filament radius through the Plateau–Rayleigh criteria (*R* = *γ*/*σ*_y_, where *R* is the minimum filament radius). High surface tension or low yield stress promotes necking and droplet formation, while the opposite combination can yield uneven or distorted filaments [53]. This relationship should be interpreted as a simplified stability criterion rather than a universal design rule, because local bath yielding, ink‐bath miscibility, crosslinking kinetics, and transient deposition dynamics can alter filament stability. Surface tension is tuned through solvent composition or surfactants [54] and quantified using a tensiometer [55] or estimated from known material properties [56]. In vascular applications, controlling interfacial stability is essential, which can reduce wall roughness and support physiological flow.

#### 2.1.5 Transparency and Biocompatibility

Optical transparency and biocompatibility support the verification and preservation of encoded architecture during fabrication and early culture, ensuring that printed structural features are retained through the onset of biological maturation. Optical transparency (typically quantified by transmittance over 300–800 nm [57, 58]) facilitates real‐time observation of nozzle position and filament formation [57]. In photo‐crosslinkable systems, it also determines the accuracy of light penetration during curing. Transparent baths such as cellulose nanofiber or gelatin methacryloyl (GelMA)‐based baths support hybrid strategies that combine EB with spatially patterned photopolymerization [59, 60], thereby improving structural precision and complexity.

Because the bath contacts cells throughout the printing process, it must maintain viability and preserve essential cellular functions. Biocompatibility is evaluated either by culturing cells directly within the bath [60] or by assessing viability of constructs printed inside the bath over several days [48]. In both cases, cell viability, proliferation, and metabolic activity are monitored over several days, often up to seven. Shorter assessments (24 h) provide early indications of cellular tolerance, while longer evaluations (72 h) offer a more complete picture of cell–material interactions without effects from oxygen or nutrient gradients [61, 62]. High biocompatibility is especially important for printing parenchymal tissues, where sustained viability and correct differentiation are required.

### 2.2 Classes of Supporting Baths and Their Architectural Implications

Supporting baths in EB can be grouped by how they provide mechanical support during fabrication, including granular, continuum, and liquid‐based systems. Because each class imposes distinct mechanical constraints, they differ in the architectural features they can reliably support. This section summarizes major bath classes with an emphasis on architectural implications (Table 1).

**TABLE 1:** Supporting bath selection guide for function‐guided embedded bioprinting.

Class	Representative materials	Key properties	Suited structures	Key design variables	Typical applications
Granular baths	Carbopol (1–40 µm), PTFE (100 µm), polyelectrolyte microgel (5 µm), PVA (72 µm), FRESH v1.0 (≈40 µm)/ FRESH v2.0 (≈25 µm), agarose particles (2–11 µm), gellan gum (20–40 µm), alginate/GelMA microgels (200 µm), pollen‐derived particles (30 µm), cell‐dense bath (≈10^8^ cells/mL)	Mechanical integrity arising from particle‐particle friction and reversible rearrangement	• High‐cell‐density constructs; • perfusion‐permissive matrices; • large or thick tissues requiring nutrient and oxygen transport	• Particle size; • particle uniformity; • yield stress; • shear‐thinning behavior; • recovery rate; • residue after removal	• Vascular tubes spanning hundreds of micrometers to several millimeters [65, 66]; • bacterial cellulose formation [68, 69]; • contractile cardiac model [22, 76]; • neural spheroids [77]; • intestinal models with high cell viability [78]; • mineralized bone models which are suitable for drug delivery applications [84, 85, 86]; • biological architectures with human‐scale cell density [12].
Continuum baths	Pluronic F127, Laponite nanoclay, cellulose‐derived baths, GelMA, HA, dECM bath, alginate, κ‐carrageenan, silk fibroin, xanthan gum	Viscoelastic polymer network with continuous stress dissipation	• Smooth interfaces; • layered walls; • luminal continuity; • optically monitored fabrication; • multi‐method printing systems	• Polymer concentration; • viscosity profile; • yield stress; • recovery behavior; • transparency;	• Omnidirectional printing [10] and scaffolds [95]; • multilayered vascular tubes [18, 103, 106, 107, 108, 110]; • vascularized liver model [100]; • intestine [20, 101]; • neural niches [102]; • aligned muscle tissues [109, 111]; • monoculture and coculture tumor models [112]; • the construction of a scaled‐down heart model with internal channels [17].
Liquid baths	Immiscible systems (fluorocarbon/silicone oils), aqueous two‐phase systems (Dextran/PEG), liquid baths (crosslinking agents)	Stabilization via interfacial tension or rapid crosslinking	• Smooth channels; • hollow or perfusable lumens; • low‐viscosity inks; • low‐drag freeform deposition; • rapid interfacial or reactive stabilization	• Interfacial tension; • density mismatch; • reaction kinetics; • Viscosity ratio; • Diffusion rate; • Crosslinking speed	• Multilayered vascular tubes [122]; • endothelialized vascular channels and branched multicellular structures [116, 119].

Abbreviations: ATPS, aqueous two‐phase systems; dECM, decellularized extracellular matrix; FRESH, freeform reversible embedding of suspended hydrogels; GelMA, gelatin methacryloyl; HA, hyaluronic acid; PEG, polyethylene glycol; PTFE, polytetrafluoroethylene; PVA, polyvinyl alcohol.

#### 2.2.1 Granular Baths

Granular baths consist of jammed particles, typically larger than one micrometer, to suppress Brownian motion [37]. Their mechanical integrity arises from particle friction and geometric confinement [32]. Their intrinsic porosity enhances nutrient and oxygen diffusion, which is advantageous for multicellular constructs and the transport pathway construction, but granular baths are less effective for applications requiring ultrasmooth interfaces.

##### 2.2.1.1 Synthetic Granular Baths

Carbopol microgels, composed of polyacrylic acid particles ranging from one to forty micrometers, remain one of the most widely used synthetic granular baths [37, 63, 64]. By adjusting polymer concentration and neutralization, its yield stress can be tuned from a few pascals to more than one hundred pascals, covering the optimal range for embedded printing [37]. Carbopol baths support a wide variety of inks, including alginate, gelatin, and GelMA [7], and have produced vascular tubes across millimeter to submillimeter scales [65, 66]. However, their electrostatic swelling makes them sensitive to cationic crosslinkers, which limits compatibility with ionically crosslinked bioinks [65, 67].

Other synthetic particle systems broaden the chemical and mechanical landscape. Polytetrafluoroethylene particles (PTFE, 100 µm) [68, 69] support high oxygen permeability and have enabled bacterial cellulose formation and large‐scale vessel fabrication [68, 69]. Polyelectrolyte microgels (5 µm) [39, 70, 71] and polyvinyl alcohol (PVA, 72 µm) [72] particles can be prepared with anionic, cationic, or zwitterionic surface chemistries, revealing a strong dependence of cell growth on charge type. These examples highlight how chemical composition governs the interaction between bath mechanics and biological performance.

##### 2.2.1.2 Biopolymeric Granular Systems

Gelatin microparticle baths introduced through the freeform reversible embedding of suspended hydrogels (FRESH) technique brought cytocompatibility and accessibility to EB. Early versions employed irregular particles (≈40 µm) [13, 73, 74], whereas the second generation used chemically synthesized particles with uniform size distributions around 25 µm [9, 22, 75], yielding smoother filament interfaces and sub‐hundred‐micrometer resolution [61]. These baths have supported the fabrication of complex tissues including contractile cardiac structures [22, 76], neural spheroids [77] and intestinal models [78], all with high cell viability. Their main limitation is the thermal sensitivity, which restricts long printing sessions and compatibility with thermosensitive inks [79].

To overcome the thermal instability of gelatin‐based supporting baths, agarose and gellan gum microparticles have been introduced as thermally stable alternatives [79, 80]. Agarose particles (2–11 µm) allow stacking of thin filaments with controlled fiber orientation, enabling meniscus‐like constructs that direct cell alignment [81, 82]. Gellan gum (20–40 µm) microgels support high‐resolution collagen (≈25 µm [83]) printing and mineralized bone models suited for drug delivery studies [84, 85, 86], although reduced optical transparency and residual particles can complicate visualization and post‐printing removal [87].

Recent developments illustrate a shift toward function‐tailored granular baths. Microfluidically produced alginate or GelMA microgels (200 µm) provide consistent particle size and tunable rheology [25]. Pollen‐derived particles (30 µm) offer natural biocompatibility and unique surface functionalities relevant for cell adhesion [88]. Cell‐dense baths (≈10^8^ cells/mL) integrate living components into the supporting bath and enable co‐printing of spheroids and perfusable networks, attaining architectures that approach human‐scale cellular density [12]. These innovations position granular baths as active contributors to tissue development rather than inert mechanical scaffolds.

Granular baths are suitable for high‐cell‐density or transport‐demanding constructs, where permeability and nutrient exchange are prioritized. Their main limitation is particle‐induced interfacial roughness and possible residue, which can compromise applications requiring ultrasmooth lumens, optical clarity, or precise barrier interfaces.

#### 2.2.2 Continuum Baths

Continuum baths consist of continuous polymer networks rather than discrete particles. Mechanical integrity is governed by molecular entanglement [10], reversible crosslinks [16], or nanofiller interactions [89]. They form continuous viscoelastic networks that provide homogeneous mechanical support, favoring architectures that require smooth interfaces, layered organization, and luminal continuity.

##### 2.2.2.1 Synthetic Gels

Pluronic is a representative thermoresponsive continuum bath. It remains fluid at low temperature and forms a viscoelastic network near room temperature through micellization. This allows omnidirectional printing, although rapid viscosity recovery may trap voids [10, 90]. Additives such as polyethylene glycol (PEG) [91] or nanoclay [52, 62, 92] extend the recovery time and broaden usable rheological ranges. Laponite nanoclay suspensions form yield stress networks at low concentrations (2%) through electrostatic interactions [89, 93]. These baths support both hydrophilic and hydrophobic inks and have been used to print branched tubular structures [45, 94] and mechanically robust scaffolds [95]. These examples demonstrate how supramolecular interactions, rather than particle jamming, can provide printable viscoelasticity.

##### 2.2.2.2 Biopolymer‐Based and Hybrid Networks

Cellulose‐derived baths exploit renewable sources and inherent transparency [57, 96]. Cellulose nanofibers and nanocrystals combine high optical transmittance with sufficient yield stress to support stable tubular structures [57], and dehydration allows transition into microfluidic templates [97]. Blending cellulose with Pluronic or other polymers increases rheological control and has supported fabrication of functional blood vessels [33, 48, 98, 99] and a vascularized liver model [100].

GelMA baths merge excellent biocompatibility with photo‐curable functionality [49]. Their transparency allows the integration with light‐based volumetric printing [60], while mild processing conditions preserve cell viability >90% [60]. EB within GelMA baths has generated large‐scale tissues such as intestinal villi [101], neural niches [102], and multilayered vascular tubes [103]. Likewise, hyaluronic acid (HA) based systems, modifiable through host–guest interactions [14, 16, 104] or bisphosphonate crosslinking [105], achieve sub‐hundred‐micrometer resolution [47] and can function as either supportive or sacrificial baths due to controlled enzymatic degradation.

In addition, decellularized extracellular matrix (dECM) offers a biocompatible option for constructing multilayered vascular models [18, 106, 107, 108] and intestinal models [20]; alginate baths support 50 µm resolution printing to enable myoblast alignment [109] and cell‐only printing [110]; κ‐carrageenan supports the printing of GelMA/PEG bioinks for muscle cell alignment [111]; silk fibroin enables cell‐laden collagen printing for monoculture and coculture tumor models [112]; and xanthan gum supports printing with feature sizes down to approximately 10 µm and enables the fabrication of miniaturized cardiac models with embedded internal channels [17]. These systems demonstrate how continuum baths can provide both mechanical fidelity and biological specificity, functioning as temporary instructive niches during early tissue formation.

Continuum baths are preferable for architectures requiring smooth interfaces, layered walls, luminal continuity, or optical monitoring, such as vascular, epithelial, and tubular models. Their homogeneous viscoelastic networks reduce particle‐induced roughness and improve interfacial definition. However, their lower permeability can limit long‐term culture of thick or highly cellular constructs unless perfusion or sacrificial transport pathways are incorporated. Rapid recovery or high viscosity may also trap voids and increase sensitivity to printing speed, extrusion rate, and bath removal conditions.

#### 2.2.3 Liquid Baths

Liquid baths stabilize printed filaments through interfacial or reactive mechanisms, enabling the formation of smooth interfaces and patent luminal spaces. Their primary functional influence arises from establishing continuous transport pathways, such as perfusable channels, while providing limited control over bulk cellular organization or spatial heterogeneity.

##### 2.2.3.1 Immiscible Systems

Printing aqueous inks into immiscible oils or fluorocarbon liquids produces interfacial confinement governed by surface tension [113]. Filament stability depends on the balance between viscous and capillary forces, often expressed through the capillary number and viscosity ratio (*η*_ink_/*η*_bath_) [114]. Interfacial jamming by nanoparticles or surfactants can create an elastic shell that suppresses capillary breakup [115]. Although immiscible systems can stabilize continuous liquid filaments, limited biocompatibility and challenges in removing oil phases restrict their biomedical use.

##### 2.2.3.2 All‐Aqueous Systems

To improve biocompatibility and expand material compatibility, all‐aqueous supporting systems have been developed based on liquid–liquid phase separation, including aqueous two‐phase systems (ATPS) and coacervates [114]. ATPS form through segregative phase separation between incompatible yet water‐soluble components, such as PEG and dextran [31, 116, 117], producing two dilute aqueous phases with ultralow interfacial tension (10^−^^4^–10^−^^1^ mN/m) that suppress Rayleigh–Plateau instabilities and provide extended stabilization windows for post‐printing crosslinking [118]. This strategy has been used to fabricate endothelialized vascular channels and branched multicellular structures entirely within aqueous environments [116, 119].

In contrast, coacervates arise from associative interactions between oppositely charged or weakly interacting macromolecules, yielding a dense, viscoelastic phase coexisting with a dilute aqueous phase. Polyelectrolyte coacervates, such as PDADMAC/PSS [119, 120] or hyaluronic acid‐chitosan systems [121], have enabled the fabrication of hierarchical, perfusable microchannel networks and hollow tubular structures through embedded or coaxial printing. Their cytocompatibility, adjustable viscoelasticity, and controllable disassembly support long‐term culture and make coacervates a complementary all‐aqueous strategy for constructing complex, biomimetic architectures.

##### 2.2.3.3 Reactive Stabilization

Liquid baths containing reactive species can induce immediate crosslinking upon ink deposition. Ionic baths containing CaCl_2_ form alginate gels, enabling multilayered vascular tubes [122]. Enzymatic and photochemical baths allow rapid curing in geometries defined by flow and light fields [113, 123, 124]. The resolution depends on the interplay among diffusion, density, and reaction kinetics, linking fluid mechanics directly to chemical crosslinking dynamics.

Liquid and all‐aqueous baths are advantageous for smooth lumens, hollow channels, low‐viscosity inks, and low‐drag freeform deposition. Their stabilization relies mainly on interfacial confinement, phase separation, or localized crosslinking, which makes them effective for channel‐forming applications. However, they provide weaker bulk mechanical support than solid‐like baths and are more sensitive to density mismatch, gravitational settling, and filament drift during long printing processes. Therefore, they are less suitable for highly stacked, load‐bearing, or long‐duration constructs unless density matching, rapid stabilization, or continuous crosslinking is carefully controlled.

In general, these comparisons indicate that supporting bath selection should be driven by the dominant architectural requirement of the target tissue model. Granular baths are preferable when permeability and high cellular density are prioritized, continuum baths are preferable when smooth interfaces and layered architectures are required, and liquid or all‐aqueous baths are preferable when low‐drag deposition and smooth luminal formation are the primary objectives.

## 3 Influence of Printing Dynamics on Tissue Architecture

**FIGURE 3:**
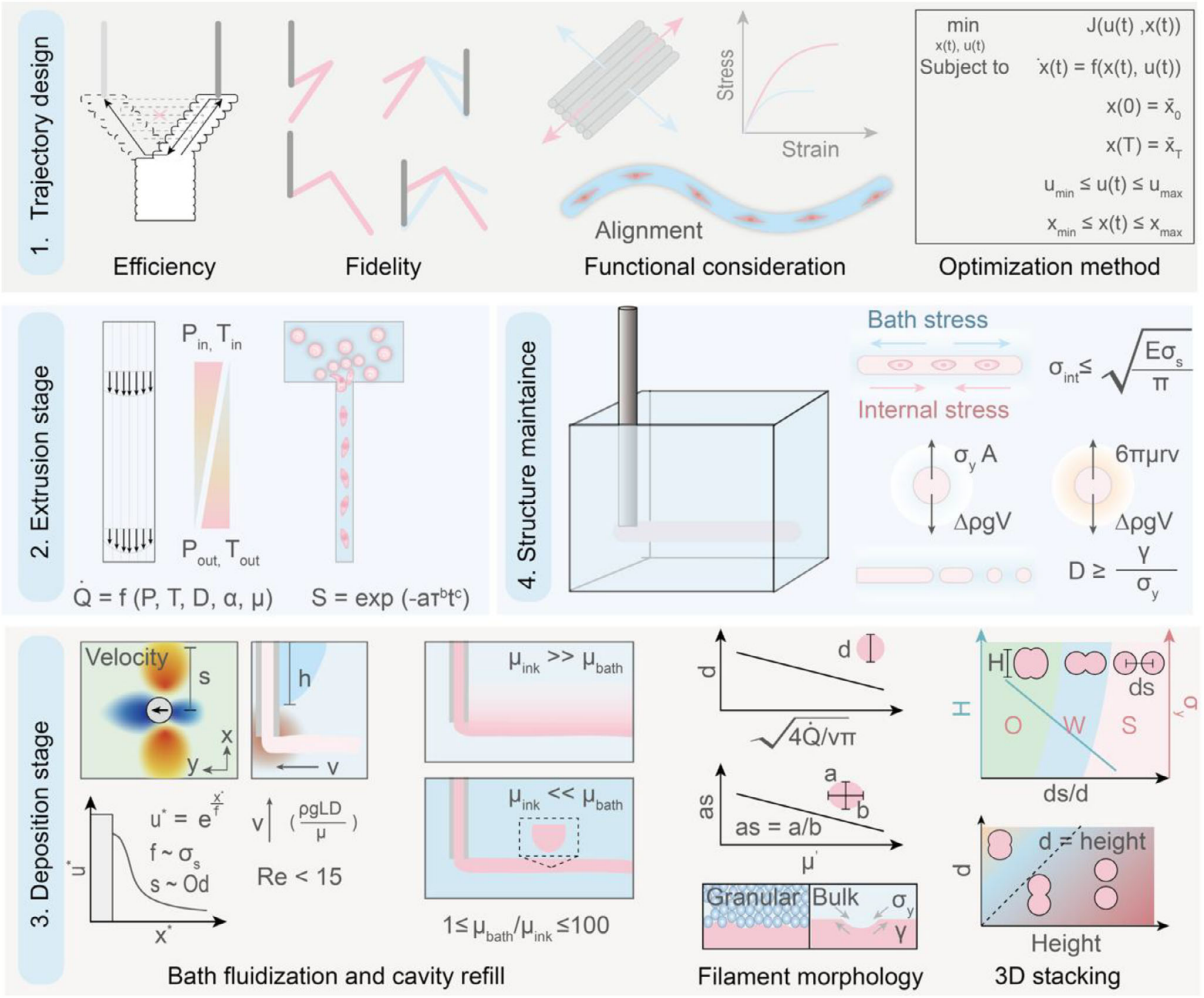
Printing dynamics as architectural encoding.

Within the proposed framework, printing processes govern how predefined structural requirements are physically encoded within the mechanical constraints imposed by the supporting bath. Decisions related to trajectory planning, extrusion control, deposition behavior, and post‐deposition stabilization govern filament morphology, interface quality, and spatial organization during fabrication. These decisions define how designed architectures emerge in practice and constrain the fidelity with which tissue‐specific structures can be constructed (Figure 3).

### 3.1 Trajectory Design as Architectural Encoding

Trajectory planning determines how individual filaments are positioned and oriented within 3D space, shaping spatial organization and anisotropy within printed tissues. The omnidirectional freedom afforded by the supporting bath expands trajectory planning beyond planar slicing, enabling curved and branched geometries [51].

Printing efficiency can be improved by minimizing non‐extrusion travel and path discontinuities, which increases throughput and helps preserve cell viability. Graph‐based methods such as skeletonization and Eulerian path optimization generate continuous toolpaths with few singularities [125], and the automated Eulerian route optimization algorithm exemplifies this by reducing interruptions in stochastic lattices [36]. Adaptive slicing further accelerates fabrication by using thicker layers in flat regions and thinner layers in high‐curvature areas [126].

Fidelity depends on the ability of the toolpath to follow complex geometries while minimizing bath disturbance. Non‐uniform rational B‐spline‐based surface generation [127] and heatmap‐guided trajectories [128] enable curvature‐aligned deposition that avoids stair‐stepping, while neural slicers and S3 slicing use machine learning to orient the nozzle along the surface normal and principal stresses, improving accuracy [129]. On the other hand, particle image velocimetry (PIV) has revealed disturbance fields caused by nozzle motion [44, 57]. Strategies to enhance consistency include tuning vertical [43] and horizontal [36] filament overlap and upward‐printing strategies [130].

Functional considerations arise from the anisotropic mechanics of filament‐based constructs [99], which typically exhibit stiffness more than twice as high along the filament axis as in the transverse direction [131]. This anisotropy can be reduced through trajectory strategies such as alternating perpendicular paths [51] or by printing low‐viscosity bioinks at narrow spacing to promote controlled coalescence [48]. Conversely, anisotropy can be exploited as a design feature [132]. Shear stresses during extrusion can align contained components, including fibrillar polymers and cells, thereby encoding directional cues within each filament [133]. This principle has enabled the fabrication of vascular [26], muscular [109], and organ scaffolds [134] in which direction‐dependent mechanics, transport properties, or contractile behavior are essential to physiological function.

Optimization integrates real‐time control and adaptive algorithms. T‐code and impedance‐responsive planning replace discrete G‐code commands with continuous, time‐aware motion control, reducing mechanical jerk and pressure fluctuations [135]. This ensures consistent filament formation, especially during high‐curvature or non‐planar movements. Future strategies also aim to couple material rheology, flow behavior, and geometric curvature in unified, feedback‐driven systems that dynamically adapt speed, pressure, and orientation for each segment of the trajectory [136].

Overall, trajectory design should be matched to the dominant architectural requirement of the target tissue. Alignment‐oriented tissues, such as cardiac and skeletal muscle, require path orientation and filament spacing that preserve anisotropy; vascular and tubular tissues require continuous, low‐interruption paths to maintain lumen patency; compartmentalized tissues require toolpaths that minimize cross‐contamination between adjacent materials or cell populations.

### 3.2 Extrusion as Flow‐Mediated Cellular and Material Control

Extrusion parameters control the flow and stress conditions experienced by bioinks during deposition, encoding filament uniformity and stress‐mediated cellular alignment while also influencing cell viability, thereby shaping architectural fidelity and cell behavior [137].

#### 3.2.1 Flow Rate Model and Shear Stress

Motor‐ and screw‐driven mechanisms regulate flow via controlled displacement but may respond slowly. Pneumatic systems allow fast modulation and maintain sterility by isolating cells from moving components. Flow rate and shear stress can be predicted using pressure–flow relationships for cylindrical [138] or conical [139] nozzles, combined with H–B or power–law constitutive models. The relationship could be expressed as *τ*_nozzle_ = 0.5*r(z)*∂*P*/∂*Z*, where *r* is the bioink radius and ∂*P/*∂*Z* is the pressure difference. To solve differential equations, the output pressure can be estimated as *P*_0_ = *ρ*_b_g*L*_B _+ *σ* + *γ/πD*_in_, where *P*_0_ is the outlet pressure, *ρ*_b_ is the supporting bath density, *g* is the gravity, *L_B_* is the immersion depth, *σ* is the stress of the bath, *γ* is the interfacial tension, and *D*_in_ is the inner diameter of the nozzle. PIV has confirmed strong agreement between these calculations and measured flow fields [140]. A recent model explicitly incorporated spatial temperature distributions for thermoresponsive inks such as GelMA, and this temperature‐dependent viscosity formulation significantly improved predictive accuracy during extrusion (*R* ^2^ > 0.9) [26]. Therefore, these models provide useful estimates of stress exposure under defined nozzle geometries and constitutive assumptions, but their accuracy depends on whether the bioink follows the selected rheological model, whether wall slip occurs, and whether temperature‐dependent viscosity or extensional flow is significant.

#### 3.2.2 Cell Viability Under Mechanical Stress

Extrusion also exposes cells to shear and extensional stresses that affect viability. High pressure [141], long residence time [139, 142], and small nozzle diameters [143] increase damage risk, and shear stresses over 100 Pa have been reported to reduce cell survival in specific extrusion‐based systems [144]. Importantly, even moderate stresses can become lethal when cells experience abrupt velocity gradients, particularly near regions of steep nozzle taper [142, 145], where extensional stresses dominate. Cell survival (*S*) can be approximated using empirical models such as *S = exp(*−*aτ^b^t^c^)*, where *a*, *b*, and *c* are fitted coefficients, and *t* is the duration [146]. This model is useful for comparing relative stress exposure within a defined printing system, but it does not fully capture extensional stress, abrupt velocity gradients, cell‐type‐specific mechanosensitivity, or stress relaxation in viscoelastic bioinks. Mitigation strategies focus on reducing peak stress and smoothing stress transients. For instance, conical nozzles reduce peak stress by smoothing velocity gradients [147], and viscoelastic inks act as natural shock absorbers that dissipate transient stress, which could protect cells [142].

Extrusion parameters should be selected according to whether the priority is cell alignment, cell survival, or dimensional accuracy. Higher shear or narrower nozzles may promote alignment in muscle or vascular smooth muscle constructs, but they can reduce viability in sensitive cell types. For cell‐dense parenchymal models, lower stress exposure and shorter residence time should be prioritized, whereas vascular and tubular models require tighter control of flow rate and nozzle diameter to maintain uniform wall thickness and lumen size.

### 3.3 Deposition as an Interface‐Encoding Process

Deposition behavior governs how extruded filaments interact with the supporting bath and with previously printed features. Deposition parameters regulate filament morphology as well as filament–filament interactions, thereby defining interface continuity, feature discreteness, and lumen smoothness. These architectural features are central to transport, barrier, and mechanical functions in printed tissues.

#### 3.3.1 Bath Fluidization and Cavity Refill

As the nozzle moves, it fluidizes a localized zone within the yield‐stress bath, generating transient crevasses that must be refilled rapidly to encapsulate the filament. The refill process depends on printing depth *h* and bath yield stress, following *σ *≤ *ρ*_b_ *gh*, indicating that higher printing velocity can promote crevasse closure by shearing the bath. However, excessive velocity induces wake formation (Reynolds number > 10) and undermines stability [148, 149]. Disturbances decay exponentially from the nozzle surface, described by *u/U = *exp(−*l/fD)*, where *u* is the local velocity, *U* is the printing velocity, *l* is the distance from the nozzle, *D* is the nozzle diameter, and *f* is the fitted value related to bath yield stress [44]. The Oldroyd number (*Oh* = *σ*_y_ *D^n^*/*KU^n^* [44]) provides a dimensionless measure of stability, with larger *Oh* associated with reduced disturbance and improved deposition precision.

Crevasse formation also highlights the importance of matching bath and ink viscosity. A highly viscous bath combined with a low‐viscosity ink resists liquefaction and refills slowly, leaving overflowed vertical trails and reducing vertical accuracy. A low‐viscosity bath paired with a high‐viscosity ink promotes intermixing, blurring filament boundaries. Empirically, maintaining a bath‐to‐ink viscosity ratio between 1 and 100 provides sufficient confinement while enabling clean filament deposition, balancing stability with interface sharpness [33].

#### 3.3.2 Filament Morphology

At the filament level, aspect ratio, diameter, and surface smoothness may govern lumen topology, mechanical anisotropy, and cellular alignment. The aspect ratio is controlled by bath yield stress [43, 57] and printing velocity [40, 150], and was reported to be linear with the transient bath viscosity *µ*_t_, where lower *µ*_t_ favors rounder cross‐sections [33]. When filament cross‐sections become approximately circular, the filament radius *R* follows mass conservation, *R = (Q̇/Uπ)^1/2^*, indicating that synchronized control of flow rate *Q̇* and nozzle speed *U* is necessary for uniform filament size along printing paths [45, 151]. Surface smoothness is strongly influenced by bath microstructure: dense continuum baths [57] and granular gels with small particles [9, 152] suppress edge waviness, whereas coarse granular baths leave surface scallops that translate into irregular luminal walls. Additionally, maintaining a balance between the extrusion flow rate and printing velocity could also refine the edge quality [45], which is especially critical for perfusable structures produced by sacrificial printing.

#### 3.3.3 Filament Coalescence and 3D Stacking

During 3D stacking, the transition from discrete filaments to continuous architecture depends on controlling overall height, horizontal fusion, and vertical integration. Overall height is primarily dictated by the printing distance: distances that are too short compress filaments and induce local over‐stacking, whereas distances matched to filament diameter weaken interlayer bonding; slightly smaller distances yield continuous sheets but can slightly increase the layer height [153]. Horizontal coalescence is set by lateral spacing and bath yield stress: tighter spacing and higher yield stress (around 10 Pa threshold in a reported system) amplify the pressure drop induced by nozzle motion, drawing adjacent filaments together, whereas low yield stress or large spacing results in partial or absent fusion [33]. Vertical coalescence depends on matching layer height with filament diameter. Layer heights below filament diameter cause excessive overlap that disrupts morphology and cell alignment, while heights comparable to filament diameter preserve anisotropic cues. Excessively large heights lead to delamination and compromised structural integrity [26].

The deposition parameters should be tuned according to the required interface state, while their quantitative applicability depends on bath rheology, ink viscosity, nozzle geometry, printing depth, crosslinking kinetics, and the time scale of bath recovery. Discrete filaments are useful for anisotropic muscle‐like architectures, controlled coalescence is needed for continuous walls or layered tissues, and smooth merging is essential for perfusable vascular or epithelial lumens. In practice, lateral spacing, layer height, printing speed, and bath‐to‐ink viscosity ratio should be adjusted together to control whether deposited features remain separated, fuse into sheets, or form continuous hollow structures.

### 3.4 Structural Persistence Before Crosslinking

Following deposition and before crosslinking, the construct enters a mechanically vulnerable period during which cellular forces, density mismatches, and interfacial phenomena may distort the intended geometry. Structural maintenance during this window determines whether the microarchitecture encoded during printing survives into maturation.

#### 3.4.1 Horizontal Stability

Horizontal stability is governed by the competition between cellular contractile forces and the resisting friction provided by the bath [110]. Cell‐mediated contraction begins shortly after printing. In one reported system, maintaining filament morphology required a storage modulus above 3.4 Pa and a yield stress exceeding 1.95 Pa, alongside limiting cellular volume fraction below 0.03. These thresholds preserve filament integrity and cellular alignment, ensuring that mechanically encoded cues are not lost before stabilization [71].

#### 3.4.2 Vertical Stability

Vertical drift arises from density differences between ink and bath. The condition *Aτ*_y_ ≥ Δ*ρVg* describes the requirement for yield stress‐dominated baths to counteract density differences between ink and bath, where *A* is the hydrodynamic surface area, and Δ*ρ* is the density difference between the bath and bioink [154]. In yield‐stress‐free baths, viscous resistance becomes the main stabilizing force, expressed by 6π*µ*_bath_ *r*_ink_ *v*_ink _≥ Δ*ρVg*, where *µ*_bath_ is the viscosity of the bath, *r*_ink_ is the radius, and *v*_ink_ is the moving velocity. Although increasing bath viscosity slows drift, yield stress remains the most reliable mechanism for maintaining structural accuracy.

#### 3.4.3 Local Stability

Local stability is shaped by interfacial tension and osmotic effects. Elevated interfacial tension can trigger Plateau–Rayleigh breakup of cylindrical filaments into droplets [155], compromising filament continuity and structural fidelity. Osmotically driven water flux can also shrink or swell filaments, offering a mechanism for controlled post‐printing adjustment of filament diameters [47], but potentially distorting fine features if unmanaged.

In short, structural persistence should be evaluated according to the failure mode most relevant to the intended construct. Cell‐dense or contractile tissues require resistance to cell‐mediated contraction, long or suspended channels require control of buoyancy and vertical drift, and fine filaments or hollow lumens require suppression of interfacial breakup and osmotic deformation. These considerations should guide bath yield stress, density matching, crosslinking timing, and culture conditions before the printed architecture is stabilized.

Taken together, across these processes, printing dynamics define the mechanisms by which architectural intent is encoded into physical structure. Trajectory design governs spatial organization and anisotropy, extrusion controls filament integrity and cell‐scale cues, deposition determines interface quality and structural integration, and structural persistence dictates whether these features are retained. These performance outcomes translate directly into the structural conditions that underpin tissue function, including how cells are positioned, how biological interfaces are maintained, how transport pathways are established, and how heterogeneity is distributed across length scales (Table 2). Within a function‐guided framework, deliberate coordination of printing dynamics becomes essential for reproducibly generating structures that support physiologically meaningful function.

**TABLE 2:** Printing dynamics as architectural encoding.

Stage	Key features	Governing methods	Functional implications	Limitations	References
Design	Efficiency	Graph‐based path optimization and adaptive slicing	Maintains structural continuity and reduces weak points that may compromise perfusion, contraction, or barrier integrity.	Algorithm performance depends on geometry complexity, nozzle access, and allowed path discontinuities.	[36, 125, 126]
Design	Fidelity	NURBS‐based trajectories, heatmap‐guided trajectories, and neural slicers/S3 slicing	Preserves curvature and surface smoothness required for stable flow, shear sensing, and interface‐dependent functions.	Requires accurate geometric input and may not account for bath deformation during nozzle motion	[44, 57, 127, 128, 129]
Design	Functional consideration	Anisotropy of filament, and shear stress‐induced alignment	Encodes anisotropy for direction‐dependent mechanics, transport, and contractile behavior.	Less suitable when isotropic mechanics, homogeneous cell distribution, or uniform diffusion is required	[26, 51, 109, 133, 134]
Design	Optimization	T‐code/impedance‐responsive planning, and feedback‐driven systems	Improves reproducibility of complex, non‐planar, or high‐curvature architectures.	Depends on sensor accuracy, control latency, model quality, and integration with extrusion pressure or flow‐rate control	[135, 136]
Extrusion	Shear stress and flow rate	*τ = 0.5r(z)∂P/∂z* *(P_0_ = ρβgLβ + σ + γ/(πD_i__n_))*	Controls filament uniformity, wall thickness, lumen size, and stress‐mediated cellular organization.	May ignore wall slip, extensional flow, and temperature‐dependent viscosity	[26, 138, 139]
Extrusion	Cell survival	*S = exp(*−*a τ^b^ t^c^)*	Preserves viable cells for subsequent maturation and functional development.	Empirical and cell‐type‐dependent	[146]
Deposition	Deposition stability	*u/U = exp(*−*l/(fD)); Oh = σᵧD^n^/(KU^n^); Re = ρvd/µ*	Protects fine channels, thin walls, and spatial compartments from blurring or erosion.	Parameters are bath‐specific and sensitive to nozzle motion, bath recovery, and printing depth.	[44]
Deposition	Filament morphology	*r = a × µt + b* *R = (Q̇/(Uπ))^1^/^2^*	Defines pore size, wall thickness, lumen topology, and local cellular microenvironment.	Deviations occur with interfacial instability or incomplete bath recovery	[33, 45, 151]
Deposition	Multi‐filament fusion	d^*^ = m × l_s_/W	Regulates wall continuity, interlayer bonding, permeability, and mechanical anisotropy.	Depends on filament spacing, layer height, yield stress, ink‐bath viscosity ratio, and crosslinking timing	[33]
Maintenance	Local stability	*R = γ/σ_y_*	Determines the minimal stable filament radius.	Affected by local yielding, ink‐bath miscibility, crosslinking, and transient capillary effects	[155]
Maintenance	Vertical stability	*Aτ_y_ ≥ ΔρVg* *6πµ_bath_r_ink_v_ink _≥ ΔρVg*	Prevents drift and sagging to maintain channel patency, stratification, and perfusion continuity.	Depends on density mismatch, bath yield stress, viscosity, construct size, and printing duration	[154]
Maintenance	Horizontal stability	*σ_int_ = (Eσ_y/_π)^1^/^2^*	Preserves alignment, interfaces, and anisotropy during early cell‐mediated remodeling.	Affected by cell density, contractility, matrix stiffness, and culture time	[71, 110]

Abbreviations: H–B, Herschel–Bulkley; Oh, Oldroyd number. Symbols are defined in the corresponding text sections; PIV, particle image velocimetry; Re, Reynolds number.

## 4 Tissue‐Specific Architectural Requirements and Functional Benchmarks

**FIGURE 4:**
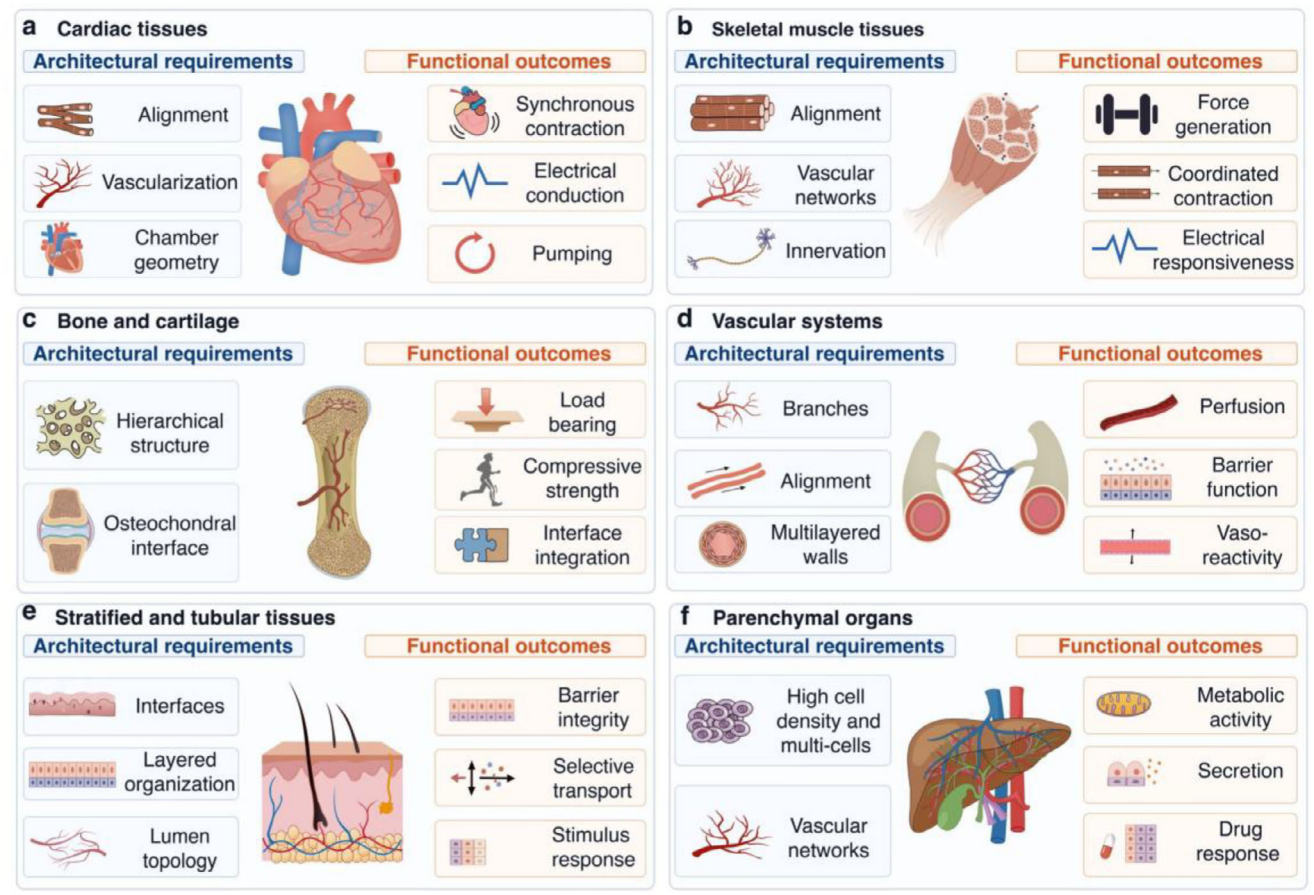
Tissue‐specific architectural requirements and functional outcomes in embedded bioprinting. Representative tissue systems are organized according to the architectural features required to support tissue‐specific function. (a) Cardiac tissues require cellular alignment, vascularization, and chamber geometry to support synchronous contraction, electrical conduction, and pumping. (b) Skeletal muscle tissues rely on aligned muscle fibers, vascular networks, and innervation for force generation, coordinated contraction, and electrical responsiveness. (c) Bone and cartilage require hierarchical structures and osteochondral interfaces to achieve load‐bearing, compressive mechanical performance, and interface integration. (d) Vascular systems require branched geometries, cellular alignment, and multilayered walls to support perfusion, barrier function, and vasoreactivity. (e) Stratified and tubular tissues depend on defined interfaces, multilayered organization, and lumen topology for barrier integrity, selective transport, and stimulus response. (f) Parenchymal organs require high cell density and vascular networks to sustain metabolic activity, secretion, and drug response.

Across tissue systems, physiological function depends on a set of basic architectural features, including cellular organization, interface continuity, transport pathways, and spatial heterogeneity. These requirements are not expressed in a uniform manner across organs, but instead take tissue‐specific forms that reflect functional demands. For example, coordinated cellular organization manifests as highly ordered cell orientation and anisotropy in cardiac and skeletal muscle, whereas in parenchymal organs it is defined by extreme cellular packing density and multicellular aggregation. Similarly, transport pathways may correspond to perfusable vascular networks in metabolically active tissues or to short‐range diffusive architectures in thin or avascular systems. Together, these requirements define tissue‐specific structural constraints that guide printing design and determine how fabrication strategies must be selected and evaluated. EB offers a means to establish such foundational architectural conditions during fabrication, yet its effectiveness depends on how these constraints are prioritized for specific tissue functions. Accordingly, tissue‐specific architectural requirements should be regarded not only as fabrication targets but also as design inputs that guide subsequent tissue maturation. Alignment provides directional cues for muscle and vascular remodeling, lumen continuity supports endothelial and epithelial barrier formation, spatial gradients direct region‐specific matrix deposition, and perfusable transport pathways sustain high‐cell‐density tissues throughout functional maturation. To make functional comparison across tissue systems more explicit, this section distinguishes three levels of evidence: architectural fidelity, biological maturation, and direct functional validation. Architectural fidelity refers to the reproduction of tissue‐relevant structures, including geometry, alignment, lumen continuity, layering, and spatial heterogeneity. Biological maturation refers to cell organization, phenotype maintenance, junction formation, sarcomere organization, extracellular matrix deposition, and lineage‐specific marker expression. Direct functional validation refers to measurable tissue‐specific behaviors, such as contraction, conduction, permeability, vasoreactivity, mechanical loading, tissue‐specific secretion, transport selectivity, and drug response. In this section, we examine how EB has been applied across representative tissue classes, focusing on the architectural features required for function, how they are encoded during fabrication, and where key limitations remain (Figure 4; Tables 3 and 4).

**TABLE 3:** Functional outcomes of embedded bioprinting across tissue types.

Tissues	Architectural fidelity	Biological maturation	Functional validation	Representative reported outcomes
Cardiac tissues	• Chamber geometry; • valve‐like structures; • helical or lamellar fiber alignment; • perfusable channels	•Cardiomyocytealignment; • sarcomere organization; • Ca^2^^+^ propagation; •electromechanical coupling	• Contractile amplitude; • pacing response; • conduction velocity; • chamber deformation; • beating stability under perfusion	• The overall dimensions of the heart are 81.31 × 64.14 × 53.59 mm^3^, with a relative error of <3% for key structures [156]. • Maintain 96% cell viability [9] and develop 1.31–2 cm/s Ca^2^^+^ conduction, 14% systolic wall thickening [9]. • Multiscale collagen hearts with 28 mm valves sustain >40 mmHg transvalvular pressure [9]. • Neonatal‐sized collagen hearts enable the reproduction of intraventricular trabecular structures and ventricular septal grid‐filling patterns [9]. • Cardiac tubes respond to 1–2 Hz pacing with anisotropic Ca^2^^+^ wave propagation [22].
Skeletal muscle tissues	• Long‐range filament alignment; • myofiber bundle geometry; • tendon interfaces; • microchannels	• Myotube formation; • myogenic marker expression; • fiber maturation; • matrix remodeling; • viability	• Force generation; • electrical stimulation response; • force transmission;	• Both the number of capillaries (increased by approximately three times) and the density of capillaries (increased by approximately four times) significantly increased compared to controls [157]. • EB‐guided architectures produce anisotropic alignment with OOP values 0.6–0.8, highest in parallel‐fiber constructs [158].
Bone and cartilage	• Pores; • stiffness gradients; • zonal architecture; • interfaces	• Osteogenic or chondrogenic marker expression; • mineralized matrix deposition; • cartilage‐specific extracellular matrix formation; • region‐specific remodeling	• Compressive or shear modulus; • interface stability; • load‐bearing response; • long‐term mechanical durability under loading	• Zonal stiffness reproduced with storage modulus increasing from cartilage to bone [84]. • Trabecular‐mimetic collagen scaffolds show 3% porosity, close to the value of 3.5% [159]. • Intervertebral disc analogues show aligned collagen rings integrating into polysaccharide‐rich nucleus pulposus [160]. • Region‐specific osteogenesis achieved through localized cue deposition [161]. • Composites achieve 7 ± 0.8 MPa compressive strength, within cancellous bone range (0.1–16 MPa) [152].
Vascular systems	• smooth lumen; • continuous channels; multilayered wall; • branched hierarchy; • controlled wall thickness	• endothelial coverage; • tight‐junction formation; • VSMC alignment; • matrix deposition	•permeability; • shear responsiveness; •vasoconstriction; vasorelaxation; • perfusion stability; • barrier function under flow	• Free‐form channels (Y‐branches, pyramids, helices) verified perfusable by dye tracing [116]. • EC monolayers reach 175% proliferation in 7 days [116]; endothelialized channels reduce 70 kDa dextran diffusion by 30% [116]. • Circumferential alignment yields ≈1.9× higher radial contractile force [26]; norepinephrine‐induced contraction reaches 6.9% ± 1% vs. 3.7% ± 1% in disordered controls [26].
Epithelial and tubular tissues	• Layered epithelium; • lumen topology, villus or fold‐like structures; • smooth interfaces	• Epithelial polarity; • tight‐junction formation; • region‐specific differentiation; • stromal interaction	• Barrier integrity; • permeability; • transport selectivity; • response to microbial or chemical stimulation	• Skin constructs exhibit clear tri‐layer organization with vascular lumens showing 2.5 µm roughness [162]. • Layer‐specific moduli tuned via collagen/pectin ratios replicate mechanical gradients [163]. • Promotes the generation of skin accessory structures such as hair follicles and sebaceous glands at the wound site [64]. Printed corneas achieve 54.7 µm roughness, with dynamic layer‐height correction minimizing tilt errors [156]. • Size‐matched villi exhibit physiological diffusion gradients and barrier function under physiological perfusion [2].
Parenchymal organs	• High cell density; • spatially organized spheroids or organoids; • perfusable microchannels; • multicellular compartments	• Phenotype maintenance; • tissue‐specific marker expression; • matrix remodeling; • vascular interaction; • long‐term viability under perfusion	• Metabolic activity; • drug response; • transport function; • tissue‐specific secretion	• Pancreas: The printed spheroids exhibited a 21.6‐fold increase in α‐SMA‐positive stromal area over 5 days and showed enhanced drug resistance, with a gemcitabine IC_50_ of 97.9 µm compared to 60.7 µm for mono‐spheroids [50]. • Liver: Achieved 1.7 × 10^8^ cells/cm^3^, matching native liver density and maintained 83% viability post‐printing [164]. • Kidney: Dual‐tubule systems form epithelial monolayers, mature endothelial lumens, and selective inulin impermeability [165].

Abbreviations: EB, embedded bioprinting; EC, endothelial cell; VSMC, vascular smooth muscle cell.

**TABLE 4:** Function‐guided design matrix for embedded bioprinting.

Target tissue function	Required architecture	Supporting bath and bioink strategy	Key printing parameters	Maturation and functional validation
Cardiac contraction and conduction	Aligned cardiomyocytes, chamber geometry, perfusable transport pathways	Granular or continuum bath; cardiac dECM bioinks; rapid crosslinking to preserve alignment and chamber shape	Filament spacing, path orientation, extrusion shear, flow rate, crosslinking time	Contractile amplitude, Ca^2^^+^ conduction, pacing response, sarcomere organization, beating stability
Skeletal muscle force generation	Myofiber alignment, bundle continuity, vascular support	Granular or continuum bath; anisotropic hydrogel or fiber‐guided bioink; matrix allowing myogenic maturation	Filament direction, filament spacing, extrusion shear, construct length, post‐print culture	Myotube alignment, fusion index, force generation, electrical stimulation response
Vascular barrier and tone	Smooth lumen, endothelial layer, multilayered wall, VSMC orientation	Continuum or all‐aqueous bath; sacrificial, coaxial, or multimaterial EB strategy; EC‐ and VSMC‐laden bioinks	Lumen diameter, lumen roughness, wall thickness, layer height, flow rate, cell orientation	Endothelial coverage, permeability, shear responsiveness, vasoconstriction, vasorelaxation
Bone and cartilage mechanical integration	Stiffness gradient, mineralized domains, zonal collagen orientation, osteochondral interface	Granular or continuum bath; calcium phosphate, collagen, GelMA, or composite bioinks; region‐specific crosslinking or mineralization	Pore size, filament spacing, material gradient, layer height, mineral phase distribution	Compressive modulus, matrix deposition, mineralization, zonal marker expression, interface stability
Epithelial and tubular transport	Continuous lumen, epithelial polarity, smooth interface, folded or villus‐like topology	Continuum or liquid bath; sacrificial or all‐aqueous EB strategy; epithelial or organoid‐laden bioinks	Wall thickness, surface roughness, channel spacing, perfusion condition	Barrier integrity, tight‐junction formation, permeability, polarity markers, transport function
Parenchymal metabolism and drug response	High cell density, spatially organized aggregates, perfusable microchannels, multicellular compartments	Cell‐dense bath, sacrificial bath, or spheroid/organoid‐laden bioink; perfusion‐compatible matrix	Cell density, channel spacing, spheroid size, perfusion rate, construct thickness	Metabolic activity, drug response, transport selectivity, long‐term viability

Abbreviations: dECM, decellularized extracellular matrix; EB, embedded bioprinting; EC, endothelial cell; GelMA, gelatin methacryloyl; VSMC, vascular smooth muscle cell.

### 4.1 Cardiac Tissues

Cardiac tissues provide a clear example in which architectural fidelity, biological maturation, and direct functional validation must be evaluated separately. At the architectural level, cardiac EB aims to reproduce chamber geometry, valve‐like structures, trabecular features, anisotropic myocardial alignment, and perfusable transport pathways. At the maturation level, these structures should support cardiomyocyte alignment, sarcomere organization, Ca^2^^+^ propagation, and electromechanical coupling. Direct functional validation further requires measurable contractile amplitude, conduction velocity, pacing responsiveness, chamber‐level deformation, and beating stability under perfusion [166, 167, 168]. In a function‐guided EB framework, these structural characteristics define the architectural targets that must be encoded during fabrication (Figure 4a).

At the anatomical scale, early demonstrations using FRESH printing of collagen and alginate established that ventricles, trabeculae, and physiological valve geometries could be fabricated with high fidelity [13, 75]. Subsequent multiscale strategies extended these capabilities to overhanging leaflets that open and close under physiological forces, indicating that EB can capture functional valve mechanics rather than static morphology [156]. These chamber‐scale constructs provide a geometric framework within which subsequent cellular organization and perfusion can be introduced, but geometry alone is insufficient to support coordinated contraction.

Reconstruction of functional myocardium requires not only correct chamber geometry but also coordinated electrical conduction and contraction. EB introduces controlled shear [169], geometric confinement [170], and functional bioinks [19] during deposition, promoting high‐density cardiomyocyte alignment (with reported cell densities reaching 3–6 × 10^8 ^cells/mL in specific systems), sarcomere organization, and synchronous contraction. By systematically varying fiber offsets and spacing, EB recreates the heart's quasi‐lamellar architecture [166, 171]. The cardiomyocyte contractile function could even drive the macro‐scale deformation to 14% wall thickness during peak systole [9]. More recently, chambered constructs containing induced pluripotent stem cell (iPSC)‐derived progenitors have shown in situ expansion and differentiation, yielding measurable electromechanical function that moves engineered tissues from morphological mimicry toward coordinated pumping [19].

Because myocardial thickness exceeds diffusion limits, perfusable vasculature is essential for sustained function. Sacrificial writing in functional tissue (SWIFT) introduces multibranched channels with diameters from approximately 400 micrometers to 1 millimeter into dense, cell‐rich matrices, supporting synchronous beating under flow [12]. Sequential printing in a reversible ink template (SPIRIT) extends this concept to soft bioinks that lack yield stress, using a reversible template to generate narrow microchannels [49]. More recent coaxial sacrificial printing integrates branched layered vessels that can be endothelialized and form functional barriers, thereby supporting and modulating cardiomyocyte contraction [25].

Collectively, cardiac EB studies have advanced from anatomical reconstruction toward electromechanical tissue models. However, the level of evidence differs across studies. Chamber geometry and valve‐like structures primarily demonstrate architectural fidelity; cardiomyocyte alignment, sarcomere organization, and Ca^2^^+^ propagation indicate biological maturation; and pacing response, contractile deformation, and perfusion‐supported beating provide more direct functional evidence. A remaining limitation is that chamber‐scale geometry, perfusable vascularization, mature force generation, and long‐term electromechanical stability are rarely achieved within one construct.

### 4.2 Skeletal Muscle Tissues

Skeletal muscle function depends on long‐range anisotropic organization and efficient force transmission. At the architectural level, EB strategies aim to generate aligned myofiber bundles, tendon‐like interfaces, and embedded transport pathways that sustain thick constructs during maturation. At the maturation level, these architectures should promote myotube formation, myogenic marker expression, fiber maturation, and matrix remodeling. Direct functional validation requires quantitative force generation, electrical stimulation response, force transmission, and fatigue‐related performance [172]. From a function‐guided perspective, EB strategies for skeletal muscle should prioritize long‐range alignment, inter‐fiber continuity, and integration with transport pathways (Figure 4b).

EB provides mechanical and geometric cues that guide myogenic alignment within soft matrices. Stress‐relaxing hydrogels with interpenetrating polymer networks have been used to support uniaxial myotube formation and functional recovery in volumetric muscle loss models [157]. A recent preprint reported FRESH‐based Embedded Bioprinting of collagen into biomimetic skeletal muscle architectures ranging from parallel to multipennate bundles, with structure‐dependent force generation under electrical stimulation [158]. High‐resolution EB [109] and shear‐guided alignment approaches [173] further demonstrate that tuning the local mechanical environment links cytoskeletal organization at the cell scale with mechanical performance at the tissue scale. Printing cell‐laden filaments into hydrogels has also enabled tendon gel integration, advancing the reconstruction of aligned muscle tendon units [21].

Analogous to the heart, thick muscle constructs require efficient oxygen and nutrient transport to sustain contractile and metabolic activity. Embedded strategies address this by incorporating vascular pathways and controlled porosity [174]. Thermally labile gelatin microparticles dispersed in supporting baths create interconnected microchannels upon melting, improving cell viability in larger constructs [175]. Muscle‐derived dECM inks printed within supporting baths promote both myogenesis and early angiogenesis, yielding tissues that show improved integration with host vasculature after implantation [176].

These studies show that EB can encode anisotropic muscle architecture and support myogenic maturation, especially through filament alignment, shear‐guided organization, and vascular‐supporting pathways. However, aligned morphology or myogenic marker expression should be interpreted as architectural or maturation evidence. Strong functional claims require quantitative force generation, stimulation‐responsive contraction, force transmission across interfaces, and sustained performance during culture or implantation. Long‐term integration with vascular and neural cues remains insufficiently validated.

### 4.3 Bone and Cartilage

Bone and cartilage tissues illustrate how spatial heterogeneity must be evaluated across distinct evidence levels. At the architectural level, EB aims to reproduce trabecular‐like pores, stiffness gradients [5, 6], mineralized domains, zonal cartilage organization, and osteochondral interfaces. At the maturation level, these structures should support osteogenic or chondrogenic differentiation, mineralized or cartilage‐specific matrix deposition, region‐specific extracellular matrix formation, and remodeling [177]. Direct functional validation requires compressive or shear mechanical testing, interface stability, and load‐bearing behavior. Within the proposed framework, these tissues impose architectural targets defined by controlled heterogeneity and graded interfaces (Figure 4c).

For bone reconstruction, supporting baths allow precise placement of calcium phosphate inks that can be strengthened after printing by ethanol or heat treatment to generate rough, osteoconductive surfaces that promote cell adhesion and osteogenic signaling [178, 179]. Spatial heterogeneity characteristic of graded bone mineralization can be introduced by printing stem cell aggregates [161], mineral‐rich collagen phases [159], or compositional patterns into defined bath regions [85]. FRESH‐based fabrication of trabecular‐like structures with millimeter‐scale pores closely reproduces cancellous morphology and supports osteogenic cell growth [159]. Beyond geometry, EB facilitates early mineralization and matrix maturation. Hydrogels containing Laponite or calcium ions enhance deposition of hydroxyapatite‐like phases [85], while printing calcium phosphate inks into cell‐laden microsphere baths mimics osteocyte‐mediated mineral nucleation through direct cell–matrix contact [180]. Aspiration‐assisted placement of human mesenchymal stem cell spheroids provides a complementary route that enhances osteogenic gene expression and remodeling via robust cell–cell cohesion [181]. Together, these strategies show how EB enables hierarchical and biologically instructive bone architectures from fibrils to organ‐scale geometry.

Cartilage function relies on zonal specialization [182]. Hybrid and direct embedded approaches enable deposition of cell‐laden hydrogels with reported stiffness ranges spanning kPa to MPa levels, generating layered constructs that recapitulate superficial, middle, and deep zones [11]. Shear applied during extrusion aligns HA fibers along the printing direction, inducing chondrocyte elongation and promoting collagen secretion, which are key to achieving tensile strength in meniscal and articular cartilage analogues [81]. Granular baths further expand design space by allowing independent tuning of microgel stiffness and interstitial filler composition, which enables induction of chondrogenesis and osteogenesis for osteochondral repair [183].

EB also supports fabrication of graded interfaces, a critical feature at the cartilage‐bone junction. Sequential printing of osteoblast‐ and chondrocyte‐laden inks into agarose or low‐acyl gellan baths yields smooth mechanical transitions that prevent delamination [84]. Similar strategies have been applied to reconstruct the intervertebral disc, printing a soft nucleus pulposus (0.3–5 kPa) analog surrounded by aligned annulus‐fibrosus fibers (≈100 kPa) that replicate native anisotropic stiffness and support region‐specific matrix deposition under hypoxic conditions [160, 184].

Overall, EB provides strong control over spatial heterogeneity in bone and cartilage models, including mineralized regions, zonal organization, and graded interfaces. These outcomes mainly establish architectural fidelity and, in some cases, matrix‐level maturation. However, geometric similarity, localized mineralization, or marker expression alone does not establish load‐bearing function. Future studies should more consistently connect printed microarchitecture with mechanical durability, interface stability, remodeling capacity, and long‐term performance under physiologically relevant loading.

### 4.4 Vascular Systems

Vascular systems require a stricter distinction between perfusable architecture and vascular function. At the architectural level, EB aims to generate smooth lumens, continuous channels, branched hierarchies, controlled wall thickness, and multilayered vessel walls. At the maturation level, these structures should support endothelial coverage, tight‐junction formation, organization of vascular smooth muscle cells (VSMCs) [185], and matrix deposition. Direct functional validation requires permeability control, shear responsiveness, vasoconstriction, vasorelaxation, and stable perfusion across branched or multilayered architectures. These features together define the structural benchmarks used to assess fabrication strategies for vascular tissues (Figure 4d).

Embedded sacrificial printing creates removable templates that form continuous channels which are subsequently lined with endothelial cells (ECs) [166]. ECs align with flow, establish tight junctions, and display shear‐responsive behavior required for vascular homeostasis. High yield stress baths, however, may hinder interfacial coalescence and leave microscale irregularities that perturb shear and impair endothelial stability [186]. Circular stacking can introduce interfacial ridges that distort lumen geometry. Aqueous‐in‐aqueous embedded systems mitigate this limitation by using low interfacial tension environments that naturally produce smooth lumens and improve endothelial adhesion and barrier function [116].

Shear stress generated during extrusion can align VSMCs. While excessive shear reduces viability, appropriately tuned conditions promote smooth muscle orientation and enhance contractility and pharmacological responsiveness [26]. Multi‐material EB can spatially pattern ECs, VSMCs and fibroblasts within a single construct, recapitulating arterial layer composition and enabling the study of cell–cell interaction, hemodynamic disturbance and targeted drug response [27].

Sacrificial printing alone can generate highly perfusable vascular trees with a confluent endothelial monolayer, but these constructs typically lack a well‐defined tunica media and adventitia. Hybrid embedded strategies that combine coaxial extrusion with cellularized supporting baths have therefore been developed to reconstruct multilayered vessel walls [187], yielding triple‐layered vascular models capable of recapitulating early atherosclerotic events [18]. Despite these advances, conventional coaxial printing remains limited in its ability to form complex bifurcations [18, 103]. Extended‐core nozzle architectures partially address this constraint by enabling continuous shell–core transitions across branch points, resulting in smoother junctions and more anatomically faithful vascular trees [25]. Beyond enabling structural fidelity, these architectures establish the mechanical and spatial cues required for endothelial maturation, barrier formation, and smooth muscle remodeling during culture. A remaining challenge, however, is that shear‐dominated extrusion aligns VSMCs predominantly along the axial direction, which contrasts with the circumferential orientation required for physiological contractility and highlights the need for strategies that decouple flow‐induced alignment from native vascular mechanics.

Taken together, vascular EB studies have progressed from perfusable channels toward endothelialized and multilayered vessel models. Perfusion capacity and lumen continuity mainly demonstrate architectural fidelity, whereas endothelial junction formation and smooth muscle organization indicate biological maturation. Direct evidence of vascular function requires barrier measurements, stable perfusion, shear‐responsive behavior, and vasoactive contraction or relaxation. Current systems still struggle to integrate smooth branching, circumferential smooth muscle alignment, endothelial barrier function, and contractile vessel‐wall behavior within one continuous architecture.

### 4.5 Epithelial and Parenchymal Organs Governed by Microarchitecture

Epithelial, tubular, and parenchymal organs depend on microscale organization, but their functional benchmarks differ. For epithelial and tubular tissues, architectural fidelity involves layered epithelium, lumen topology, folded or villus‐like structures, and smooth biological interfaces [188]. Biological maturation requires epithelial polarity, tight‐junction formation, region‐specific differentiation, and stromal interaction. Direct functional validation should include barrier integrity, permeability, transport selectivity, and response to microbial, chemical, or inflammatory stimulation. For parenchymal tissues, architectural fidelity involves high cell density, spatially organized spheroids or organoids, perfusable microchannels, and multicellular compartments. Biological maturation requires phenotype maintenance, tissue‐specific marker expression, extracellular matrix remodeling, and vascular interaction, while direct functional validation should include tissue‐specific secretion, metabolic activity, drug response, and transport function. These architectural features serve as maturation‐guiding cues that support the establishment of epithelial polarity, barrier formation, and long‐term metabolic stability during tissue development. Reproducing these tissues therefore requires precise control over microarchitectural features that constrain cell behavior.

#### 4.5.1 Stratified and Tubular Organs

Stratified tissues such as skin and cornea rely on epithelial‐stromal boundaries, micron‐scale layering and smooth interfaces that underlie barrier integrity, optical clarity and appendage morphogenesis [162, 189] (Figure 4e). In skin models, EB enables patterning of epidermal, dermal and hypodermal compartments while maintaining gradients that drive in vivo remodeling [163]. Supporting baths formulated with starch produce vascular channels with 2.5 µm‐scale luminal smoothness [162], and multiply crosslinked HA‐Pluronic inks maintain stem cell compatibility, supporting wound closure and follicle regeneration [64]. Together, these studies show that EB controls both layered tissue organization and the microenvironmental cues required for epithelial stratification and appendage formation.

Corneal models further highlight how bath microstructure can define microtopography. Printing collagen into microgel baths with different particle sizes reveals that larger microspheres (≈40 µm) promote cellular spreading, while covalently crosslinked collagen hydrogels resist contraction because of stable viscoelastic properties [190]. Refinement of layer height reduces surface roughness toward physiological values of about 6 µm, illustrating how EB can approach the optical tolerances required for transparency [156].

Tubular hollow organs such as the intestine require sculpted lumens containing villi, crypts, and circular folds. By stabilizing these delicate features during printing, embedded constructs induce regional polarization, tight junction formation, and assembled microvasculature [78]. Vertical embedded extrusion has reproduced villus and follicle morphogenesis [101], while FRESH printed compliant scaffolds have supported in vivo regeneration of villous structures [191]. Designs that incorporate controlled shear gradients and fold geometries reproduce spatial heterogeneity in epithelial, microbial and immune behavior, underscoring the principle that lumen topology acts as a biologically instructive cue [2].

#### 4.5.2 Parenchymal Organs

Parenchymal organs such as the pancreas, liver, and kidney demand dense cellular packing and integrated microvasculature. EB supports these requirements by enabling spatial placement of microvessels and cell aggregates within high‐cell‐density baths, thereby maintaining viability and metabolic function (Figure 4f).

In the pancreas, embedded positioning of β‐cell spheroids or emulsified clusters yields constructs with more precise stimulus response dynamics, including hormone secretion, than homogeneous cell distributions [60]. Pancreatic ductal adenocarcinoma models printed into viscoelastic baths preserve stromal‐epithelial interactions that contribute to native drug resistance, demonstrating how bath‐mediated stabilization can maintain complex tumor microenvironments [50].

Liver engineering has leveraged dense cell biphasic inks and removable support media to produce constructs at near physiological cell densities, around 1 × 10^8^ cells/mL, with perfusable vascular channels that enhance metabolic marker expression and vascularization [164]. Cellulose‐based removable baths facilitate hepatocyte spheroid formation and graft vascularization after implantation, linking printed architecture to functional organoid behavior [100].

Kidney reconstruction illustrates the potential of embedded approaches to encode intertwined tubular and vascular compartments. The κ‐carrageenan submicrogel baths support high‐fidelity placement of nephron‐like tubular‐vascular units with high viability [192], while proximal tubule‐on‐chip hybrids exhibit physiological solute transport and drug responses [165]. Despite this progress, recapitulating glomerular filtration and long‐term epithelial‐endothelial coupling remains a major challenge that will require finer spatial control and highly stable perfusion.

Across epithelial, tubular, and parenchymal models, EB has demonstrated strong capacity to organize cells, interfaces, and transport pathways at the microscale. However, the current evidence varies substantially across tissue systems. Layered organization, lumen topology, and high cell density mainly support architectural claims; polarity, junction formation, and marker expression indicate maturation; secretion, transport selectivity, metabolic activity, drug response, and long‐term perfusion stability provide stronger functional evidence. Future work should therefore avoid equating organ‐like geometry or dense cellular organization with mature tissue function unless supported by tissue‐specific functional readouts.

### 4.6 Cross‐Tissue Architectural Design Principles

Despite being developed for distinct biological targets and fabrication contexts, EB studies reveal several recurring architectural design principles across tissue systems. The first principle is alignment. In cardiac and skeletal muscle tissues, alignment supports anisotropic contraction, electrical propagation, and force transmission [9, 109]. In vascular systems, cellular orientation influences smooth muscle remodeling and vasoactive behavior. Therefore, filament direction, extrusion‐induced shear, and post‐print remodeling should be selected according to whether the target function depends on directional mechanics, conduction, or contractility [27].

The second principle is lumen and interface continuity. Vascular, epithelial, tubular, and parenchymal tissues all require continuous lumens or stable biological interfaces to support perfusion, barrier formation, and selective transport [116]. In these systems, lumen smoothness, wall integrity, and interfacial sharpness are not merely geometric outcomes, but functional prerequisites for endothelialization, epithelial polarity, permeability control, and flow stability [16]. Supporting bath type, particle size, interfacial tension, filament coalescence, and bath removal should therefore be evaluated according to their effects on lumen quality and interface preservation.

The third principle is spatial heterogeneity. Native tissues rarely exhibit uniform structure; instead, they depend on gradients in stiffness, composition, porosity, mineralization, cellular phenotype, and biochemical cues [182]. This is most evident in bone, cartilage, epithelial tissues, and organoid‐based parenchymal models [84], but it also applies to vascular and cardiac systems where regional geometry and wall organization regulate mechanical or transport behavior. EB is particularly useful when spatial heterogeneity must be encoded within soft or cell‐dense matrices, but the resulting gradients should be linked to region‐specific maturation or function rather than described only as patterned morphology [193].

The fourth principle is coordinated control of cell density and perfusion. High‐cell‐density constructs are essential for parenchymal, cardiac [17], and muscle tissues, but increasing cell density also increases oxygen and nutrient demand and can compromise printability [12]. Perfusable channels, sacrificial pathways, porous supporting baths, and post‐print perfusion should therefore be designed together with cell‐density targets [194]. In this sense, dense cellular organization and transport architecture should be considered coupled design variables rather than independent outcomes.

Finally, EB‐defined architectures should be interpreted as time‐dependent maturation templates rather than static printed structures [19]. The initial architecture determines cell position, orientation, confinement, interface continuity, and transport access, but these features can be progressively remodeled during culture through cell‐mediated contraction, extracellular matrix deposition, hydrogel degradation, matrix compaction, and perfusion‐induced remodeling [20]. This dynamic behavior can either reinforce the intended architecture, such as alignment maturation in muscle or endothelial barrier formation in vascular channels [116], or distort it, such as lumen narrowing, filament contraction, interface blurring, or loss of spatial gradients. Therefore, post‐fabrication maturation should be considered part of the design process: bath removal, crosslinking stability, matrix degradability, perfusion, and mechanical or electrical stimulation should be selected according to whether the printed architecture is expected to remain stable, remodel, or mature into a different functional state. Across tissues, this time‐dependent view links initial printing fidelity with later biological maturation and direct functional validation.

## 5 Challenges and Opportunities for Clinical Translation

**FIGURE 5:**
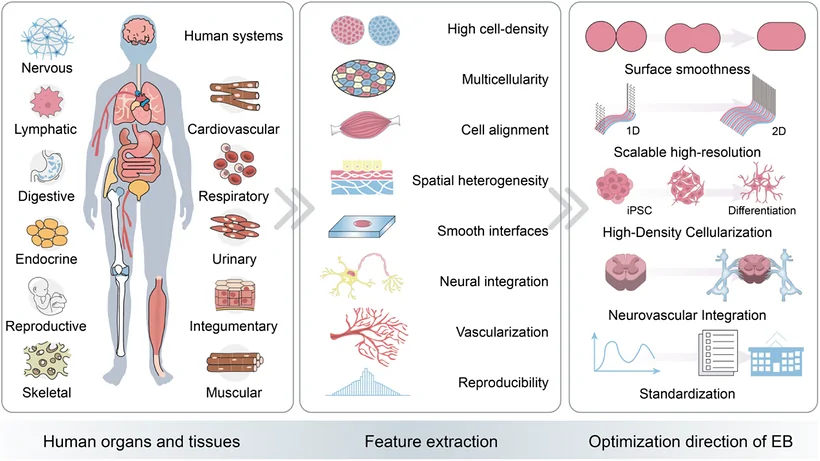
Challenges and opportunities for clinical translation.

As control over supporting bath formulation and process execution has improved, the central challenge in EB is shifting from demonstrating the feasibility of printing complex geometries toward understanding how printing‐defined architectures can be intentionally designed to support tissue‐specific function. Tissue function does not arise from fabrication techniques or materials in isolation, but from the architectural structures they establish and stabilize. EB therefore operates through a hierarchical design logic in which target tissue functions define required structural characteristics, while supporting bath mechanics and printing dynamics serve as enabling tools to encode those structures during fabrication. From this perspective, many translational limitations in EB arise from mismatches between printed architectures and the structural requirements imposed by native tissue function (Figure 5).

The translational scope of EB should therefore be considered in a staged manner. In the near term, EB is most likely to contribute to human‐relevant in vitro platforms, including disease models, drug screening systems, toxicity testing, and patient‐specific tissue models, where architectural control can improve physiological relevance without requiring full organ‐level function. In this context, success should be evaluated by whether EB constructs reproduce key tissue‐specific functions, such as barrier integrity, contractile response, perfusion‐dependent metabolism, inflammatory activation, or drug responsiveness. In the longer term, regenerative or implantable applications will require substantially higher levels of structural integration, vascular and neural coupling, mechanical durability, immune compatibility, manufacturing reproducibility, and regulatory validation. Distinguishing these near‐term and long‐term goals is important because the evidence required for a predictive in vitro model differs from that required for an implantable tissue substitute.

Native tissues feature extraordinarily smooth surfaces essential for physiological function: articular cartilage reaches roughness values around 7–8 µm [195], skin about 6 µm [196], and coronary arteries below 1 µm [197]. Reproducing such smoothness remains difficult in EB constructs because filament diameters typically exceed 10 µm and bath‐induced disturbances deform freshly deposited layers [23, 109]. Current solutions focus on minimizing inter‐filament discontinuities, including using continuum baths to prevent granular‐induced waviness, tuning interfacial tension to promote filament merging, and employing bioinks capable of rapid phase separation or chemical solidification to stabilize fine surfaces [116]. Despite these advances, achieving native‐level surface fidelity remains an open challenge tightly linked to the filamentary nature of EB.

As a filament‐based process, EB tightly couples printing resolution to fabrication time. Finer features improve smoothness and microstructural fidelity but slow printing dramatically, risking hypoxia and nutrient deprivation in cell‐laden constructs [98]. Speed‐resolution trade‐offs may be addressed through multi‐channel or microfluidic printheads that switch materials rapidly [99], as well as application‐specific resolution strategies in which microscale precision is selectively applied to tissues such as helically aligned myocardium, while coarser architectures are sufficient for applications like drug screening or bulk tissue models.

Human tissues operate at extremely high cellular densities: over 45 000 epidermal cells per mm^2^ in skin [198], more than 10^6^ ECs per mm^2^ in vessels [199], and roughly 10^8^ cells per gram in liver [200]. Clinically relevant cell numbers remain challenging to obtain [201]. Recent scaffold‐minimal approaches have demonstrated that iPSCs can assemble at ultrahigh densities approaching 10^9^ cells/mL, underscoring the extent of native cellular packing achievable in principle [202]. Increasing cell density in conventional bioinks typically reduces viscosity and structural stability, compromising printability. Within EB, sacrificial strategies offer a complementary route by spatially distributing dense cellular regions within perfusable supporting baths that sustain oxygen and nutrient transport, enabling parenchymal‐like architectures.

Beyond vascularization, most organs depend on neural regulation to coordinate contractility, secretion, sensation, and homeostasis [203]. In contrast to advances in perfusable architectures, neural integration remains underdeveloped in EB. The challenges are architectural as much as biological: innervation requires aligned conduits, permissive microenvironments, and spatial coupling with vascular and parenchymal compartments. Without incorporating neural guidance and integration into the architectural design space, many constructs will remain mechanically functional but physiologically incomplete.

These translational barriers can be further divided into EB‐specific and field‐wide challenges. EB‐specific challenges include supporting bath residue, bath removal, particle‐induced surface roughness, interfacial instability, filament drift, incomplete bath recovery, and the difficulty of preserving luminal smoothness or multilayered interfaces during long‐term culture. These issues directly affect architectural fidelity and are therefore central to EB as a fabrication strategy. In contrast, cell sourcing, batch‐to‐batch bioink variability, incomplete vascularization and innervation, limited tissue maturation, immune compatibility, storage, scalability, and regulatory approval are shared challenges across bioprinting and tissue engineering. Clarifying this distinction helps define which problems can be addressed by improving EB process design and which require broader advances in cell biology, biomaterials, bioreactors, manufacturing, and regulatory science.

In addition, these constraints expose a central integration dilemma for EB. Smooth luminal interfaces, high cellular density, anisotropic alignment, perfusable transport networks, and neural or stromal coupling are all required for functional tissue construction, yet they often impose distinct and sometimes conflicting demands on supporting bath mechanics, bioink rheology, stabilization kinetics, and maturation conditions. For example, granular baths may support dense cellular compartments and nutrient exchange but can compromise luminal smoothness; low‐drag liquid or continuum baths may improve interface quality but provide limited support for thick or highly stacked architectures; and high‐resolution printing improves local fidelity but reduces throughput and may prolong cell exposure to nonphysiological stress. Therefore, addressing this single‐construct integration dilemma requires a broader next‐generation EB ecosystem that connects fabrication, structural refinement, process control, post‐print maturation, and functional validation.

Five complementary directions might be important. First, multimaterial and microfluidic printheads can enable rapid switching, co‐extrusion, or sequential deposition of distinct bioinks, sacrificial materials, and cell populations, allowing vascular, stromal, parenchymal, and neural compartments to be spatially organized within the same construct. Second, hybrid EB strategies that integrate embedded printing with photopatterning or volumetric‐like optical approaches may help decouple macroscale shape formation from local microstructural refinement. In such systems, EB can define large‐scale anatomical geometry and perfusable architectures, while light‐based methods can refine luminal interfaces, microchannels, gradients, and alignment cues in selected regions. Third, model‐guided and feedback‐controlled printing can improve process predictability by coupling rheological characterization, computational fluid dynamics‐based analysis, machine learning, and in situ imaging to predict filament deformation, bath recovery, fusion behavior, and shape drift. These tools could support closed‐loop adjustment of flow rate, printing speed, path spacing, and crosslinking timing during fabrication. Fourth, perfusion‐maturation bioreactors can extend EB beyond structural fabrication by applying fluid shear, cyclic mechanical loading, electrical pacing, biochemical gradients, and nutrient perfusion to promote tissue‐specific maturation after printing. Fifth, standardized functional readout panels are needed to determine whether EB‐defined architectures actually support tissue‐specific function. Such panels should include contraction and conduction for cardiac tissues, permeability and vasoreactivity for vascular tissues, force generation for skeletal muscle, barrier and transport function for epithelial tissues, and metabolism, secretion, and drug response for parenchymal tissues. Together, these directions provide a route for moving EB from printable geometry toward reproducible, functionally validated tissue models.

Building on this distinction, reproducibility and regulatory clarity will require standardized reporting and validation. Variability in cell sourcing, supporting bath formulation, bioink properties, printing parameters, bath removal, and maturation protocols can all alter structural and functional outcomes. Therefore, EB studies should report a minimum set of fabrication and validation parameters, including supporting bath composition, yield stress, shear‐thinning behavior, recovery time, particle size or network type, bioink composition and rheology, cell density, nozzle geometry, nozzle diameter, immersion depth, flow rate, printing speed, path spacing, layer height, crosslinking strategy, bath removal method, culture duration, viability, structural fidelity, perfusion capability, and tissue‐specific functional assays. Establishing standardized metrics for architectural fidelity, mechanical properties, lumen or surface quality, perfusion capability, biological maturation, and direct tissue‐specific functionality will be critical. For scalable implementation, these reporting standards should also support cross‐batch reproducibility and automated process control. Key scalability‐related variables include bath preparation, particle‐size distribution, bath removal efficiency, printhead calibration, extrusion stability, path execution accuracy, and construct‐to‐construct variability. Future EB platforms will likely require automated bath preparation, integrated rheological quality control, in situ imaging, closed‐loop adjustment of printing parameters, and standardized acceptance criteria for structural and functional outputs. Manufacturing throughput may also be improved through parallelized printing strategies, multiple independently controlled printheads, automated construct handling, and integration with multiwell tissue‐model production platforms. These approaches may help increase construct‐to‐construct consistency while reducing manual handling and fabrication time, especially for drug screening and standardized in vitro testing applications. Equally important is the development of regulatory frameworks that define safety, quality control, and long‐term performance standards for bioprinted tissues, which remain insufficiently defined.

Taken together, these challenges indicate that the translational potential of EB depends less on expanding material libraries or printing modalities than on achieving consistent alignment between tissue function, architectural design targets, and the fabrication strategies used to encode them. Recent policy shifts by funding agencies and regulatory bodies, including those in the United Kingdom, the United States Food and Drug Administration, and the National Institutes of Health, are increasingly emphasizing human‐relevant, animal‐free functional in vitro platforms. In this context, EB is most powerful when applied as a design‐oriented approach that clarifies which aspects of tissue function can be directly encoded through architecture and which must emerge through post‐printing biological organization.

## 6 Conclusion

EB has evolved from a freeform fabrication technique into a design‐oriented strategy for engineering functional tissue architecture. This review advances the field by organizing EB around a function‐guided framework in which tissue function defines the structural characteristics that must be reproduced, and supporting bath mechanics and printing dynamics are selected to encode those structures during fabrication. By organizing EB around this function‐guided framework, this review clarifies how material behavior and process control converge to determine architectural fidelity and, consequently, physiological relevance. The central challenge ahead is to identify tissue‐specific architectural benchmarks that explain how printed structure gives rise to physiological function. Progress will depend on designing fabrication strategies around native structural constraints and evaluating outcomes using functional measures that reflect architecture. Meeting these goals will support the use of EB in reproducible disease models, drug evaluation platforms, and longer‐term precision tissue engineering applications.

## Author Contributions

Q.L. and H.Z. conceptualized the idea and wrote the outline of the manuscript. H.Y., Q.L., and H.Z. supervised the project. Y.C., J.L., and Q.L. researched data for the article and contributed substantially to the discussion of content and the writing of the article. All authors reviewed and approved the manuscript.

## Conflicts of Interest

The authors declare no conflicts of interest.

## Data Availability

Data sharing not applicable to this article as no datasets were generated or analyzed during the current study.

## References

[1] H. Chang , Q. Liu , J. F. Zimmerman , et al., “Recreating the Heart's Helical Structure‐Function Relationship With Focused Rotary Jet Spinning,” Science 377, no. 6602 (2022): 180–185, 10.1126/science.abl6395. 35857545 PMC10077766

[2] Z. Gao , H. Du , S. Yu , et al., “3D Bioprinted Human‐Scale Intestine Models for Physiological and Microbial Insights Through Fluid‐Driven Heterogeneity,” Science Advances 11, no. 47 (2025): ady6562, 10.1126/sciadv.ady6562. PMC12637300 41270182

[3] S. Freeman , R. Ramos , P. Alexis Chando , et al., “A Bioink Blend for Rotary 3D Bioprinting Tissue Engineered Small‐Diameter Vascular Constructs,” Acta Biomaterialia 95 (2019): 152–164, 10.1016/j.actbio.2019.06.052. 31271883

[4] E. Bosch‐Rue , L. M. Delgado , F. J. Gil , and R. A. Perez , “Direct Extrusion of Individually Encapsulated Endothelial and Smooth Muscle Cells Mimicking Blood Vessel Structures and Vascular Native Cell Alignment,” Biofabrication 13, no. 1 (2020): 015003, 10.1088/1758-5090/abbd27. 32998120

[5] S. Weiner and H. D. Wagner , “The material bone: Structure‐Mechanical Function Relations,” Annual Review of Materials Science 28, no. 1 (1998): 271–298, 10.1146/annurev.matsci.28.1.271.

[6] B. Clarke , “Normal Bone Anatomy and Physiology Clinical,” Journal of the American Society of Nephrology 3, no. 3 (2008): S131–S139, 10.2215/CJN.04151206. PMC3152283 18988698

[7] L. Ning , R. Mehta , C. Cao , et al., “Embedded 3D Bioprinting of Gelatin Methacryloyl‐Based Constructs With Highly Tunable Structural Fidelity,” ACS Applied Materials & Interfaces 12, no. 40 (2020): 44563–44577, 10.1021/acsami.0c15078. 32966746

[8] S. Santoni , S. G. Gugliandolo , M. Sponchioni , D. Moscatelli , and B. M. Colosimo , “3D Bioprinting: Current Status and Trends—A Guide to the Literature and Industrial Practice,” Bio‐Design and Manufacturing 5 (2022): 14–42, 10.1007/s42242-021-00165-0.

[9] A. Lee , A. R. Hudson , D. J. Shiwarski , et al., “3D Bioprinting of Collagen to Rebuild Components of the Human Heart,” Science 365, no. 6452 (2019): 482–487, 10.1126/science.aav9051. 31371612

[10] W. Wu , A. DeConinck , and J. A. Lewis , “Omnidirectional Printing of 3D Microvascular Networks,” Advanced Materials 23, no. 24 (2011): H178—H183, 10.1002/adma.201004625. 21438034

[11] B. A. G. de Melo , Y. A. Jodat , S. Mehrotra , et al., “3D Printed Cartilage‐Like Tissue Constructs With Spatially Controlled Mechanical Properties,” Advanced Functional Materials 29, no. 51 (2019): 1906330, 10.1002/adfm.201906330. 34108852 PMC8186324

[12] M. A. Skylar‐Scott , S. G. M. Uzel , L. L. Nam , et al., “Biomanufacturing of Organ‐Specific Tissues With High Cellular Density and Embedded Vascular Channels,” Science Advances 5, no. 9 (2019): aaw2459, 10.1126/sciadv.aaw2459. PMC6731072 31523707

[13] T. J. Hinton , Q. Jallerat , R. N. Palchesko , et al., “Three‐Dimensional Printing of Complex Biological Structures by Freeform Reversible Embedding of Suspended Hydrogels,” Science Advances 1, no. 9 (2015): 1500758, 10.1126/sciadv.1500758. PMC4646826 26601312

[14] C. B. Highley , C. B. Rodell , and J. A. Burdick , “Direct 3D Printing of Shear‐Thinning Hydrogels Into Self‐Healing Hydrogels,” Advanced Materials 27, no. 34 (2015): 5075–5079, 10.1002/adma.201501234. 26177925

[15] T. Bhattacharjee , S. M. Zehnder , K. G. Rowe , et al., “Writing in the Granular Gel Medium,” Science Advances 1, no. 8 (2015): 1500655, 10.1126/sciadv.1500655. PMC4643780 26601274

[16] K. H. Song , C. B. Highley , A. Rouff , and J. A. Burdick , “Complex 3D‐Printed Microchannels Within Cell‐Degradable Hydrogels,” Advanced Functional Materials 28, no. 31 (2018): 1801331, 10.1002/adfm.201801331.

[17] N. Noor , A. Shapira , R. Edri , I. Gal , L. Wertheim , and T. Dvir , “3D Printing of Personalized Thick and Perfusable Cardiac Patches and Hearts,” Advanced Science 6, no. 11 (2019): 1900344, 10.1002/advs.201900344. 31179230 PMC6548966

[18] G. Gao , W. Park , B. S. Kim , et al., “Construction of a Novel In Vitro Atherosclerotic Model From Geometry‐Tunable Artery Equivalents Engineered via in‐Bath Coaxial Cell Printing,” Advanced Functional Materials 31, no. 10 (2020): 2008878, 10.1002/adfm.202008878.

[19] M. E. Kupfer , W. H. Lin , V. Ravikumar , et al., “In Situ Expansion, Differentiation, and Electromechanical Coupling of Human Cardiac Muscle in a 3D Bioprinted, Chambered Organoid,” Circulation Research 127, no. 2 (2020): 207–224, 10.1161/CIRCRESAHA.119.316155. 32228120 PMC8210857

[20] J. A. Brassard , M. Nikolaev , T. Hubscher , M. Hofer , and M. P. Lutolf , “Recapitulating Macro‐Scale Tissue Self‐Organization Through Organoid Bioprinting,” Nature Materials 20, no. 1 (2021): 22–29, 10.1038/s41563-020-00803-5. 32958879

[21] D. H. Kang , F. Louis , H. Liu , et al., “Engineered Whole Cut Meat‐Like Tissue by the Assembly of Cell Fibers Using Tendon‐Gel Integrated Bioprinting,” Nature Communications 12, no. 1 (2021): 5059, 10.1038/s41467-021-25236-9. PMC8385070 34429413

[22] J. Bliley , J. Tashman , M. Stang , et al., “FRESH 3D Bioprinting a Contractile Heart Tube Using Human Stem Cell‐Derived Cardiomyocytes,” Biofabrication 14, no. 2 (2022): 024106, 10.1088/1758-5090/ac58be. PMC9206822 35213846

[23] S. Duraivel , D. Laurent , D. A. Rajon , et al., “A Silicone‐Based Support Material Eliminates Interfacial Instabilities in 3D Silicone Printing,” Science 379, no. 6638 (2023): 1248–1252, 10.1126/science.ade4441. 36952407

[24] Y. Fang , Y. Guo , B. Wu , et al., “Expanding Embedded 3D Bioprinting Capability for Engineering Complex Organs With Freeform Vascular Networks,” Advanced Materials 35, no. 22 (2023): 2205082, 10.1002/adma.202205082. 36796025

[25] P. P. Stankey , K. T. Kroll , A. J. Ainscough , et al., “Embedding Biomimetic Vascular Networks via Coaxial Sacrificial Writing Into Functional Tissue,” Advanced Materials 36, no. 36 (2024): 2401528, 10.1002/adma.202401528. 39092638

[26] Q. Li , S. Yu , Y. Wang , et al., “Programmable Embedded Bioprinting for One‐Step Manufacturing of Arterial Models With Customized Contractile and Metabolic Functions,” Trends in Biotechnology 43, no. 4 (2025): 918–945, 10.1016/j.tibtech.2024.11.019. 39779422

[27] Q. Li , S. Yu , Y. Wang , et al., “Multiscale Bioprinted Arterial Models Recapitulate Synergistic Microenvironmental Interactions in Vascular Disease,” Cell Biomaterials 2, no. 5 (2025): 100257, 10.1016/j.celbio.2025.100257.

[28] Z. A. Sexton , D. Rütsche , J. E. Herrmann , et al., “Rapid Model‐Guided Design of Organ‐Scale Synthetic Vasculature for Biomanufacturing,” Science 388, no. 6752 (2025): 1198–1204, 10.1126/science.adj6152. 40504910 PMC12490288

[29] M. Rocca , A. Fragasso , W. Liu , M. A. Heinrich , and Y. S. Zhang , “Embedded Multimaterial Extrusion Bioprinting,” SLAS Technology 23, no. 2 (2018): 154–163, 10.1177/2472630317742071. 29132232 PMC5906133

[30] X. Mo , Y. Zhang , Z. Wang , et al., “Satellite‐Based on‐Orbit Printing of 3D Tumor Models,” Advanced Materials 36, no. 34 (2024): 2309618, 10.1002/adma.202309618. 38145905

[31] M. Becker , M. Gurian , M. Schot , and J. Leijten , “Aqueous Two‐Phase Enabled Low Viscosity 3D (LoV3D) Bioprinting of Living Matter,” Advanced Science 10, no. 8 (2023): 2204609, 10.1002/advs.202204609. 36585374 PMC10015849

[32] Z. T. Xie , D. H. Kang , and M. Matsusaki , “Resolution of 3D Bioprinting Inside Bulk Gel and Granular Gel Baths,” Soft Matter 17, no. 39 (2021): 8769–8785, 10.1039/d1sm00926e. 34604877

[33] Q. Li , L. Ma , Z. Gao , et al., “Regulable Supporting Baths for Embedded Printing of Soft Biomaterials With Variable Stiffness,” ACS Applied Materials & Interfaces 14, no. 37 (2022): 41695–41711, 10.1021/acsami.2c09221. 36070996

[34] W. Hua , C. Zhang , K. Mitchell , et al., “Filament Formation Mechanisms in Yield‐Stress Fluid‐Enabled Embedded Ink Writing,” Additive Manufacturing 91 (2024): 104353, 10.1016/j.addma.2024.104353.

[35] M. E. Cooke and D. H. Rosenzweig , “The Rheology of Direct and Suspended Extrusion Bioprinting,” APL Bioengineering 5, no. 1 (2021): 011502, 10.1063/5.0031475. 33564740 PMC7864677

[36] R. D. Weeks , R. L. Truby , S. G. M. Uzel , and J. A. Lewis , “Embedded 3D Printing of Multimaterial Polymer Lattices via Graph‐Based Print Path Planning,” Advanced Materials 35, no. 5 (2023): 2206958, 10.1002/adma.202206958. 36404106

[37] C. S. O'Bryan , T. Bhattacharjee , S. L. Marshall , W. G. Sawyer , and T. E. Angelini , “Commercially Available Microgels for 3D Bioprinting,” Bioprinting 11 (2018): e00037, 10.1016/j.bprint.2018.e00037.

[38] S. Shin , L. G. Brunel , B. Cai , et al., “Gelation of Uniform Interfacial Diffusant in Embedded 3D Printing,” Advanced Functional Materials 33, no. 50 (2023): 2307435, 10.1002/adfm.202307435. 38646474 PMC11031202

[39] C. S. O'Bryan , C. P. Kabb , B. S. Sumerlin , and T. E. Angelini , “Jammed Polyelectrolyte Microgels for 3D Cell Culture Applications: Rheological Behavior With Added Salts,” ACS Applied Bio Materials 2, no. 4 (2019): 1509–1517, 10.1021/acsabm.8b00784. 35026924

[40] T. Calais , N. D. Sanandiya , S. Jain , et al., “Freeform Liquid 3D Printing of Soft Functional Components for Soft Robotics,” ACS Applied Materials & Interfaces 14, no. 1 (2022): 2301–2315, 10.1021/acsami.1c20209. 34962370

[41] M. E. Prendergast and J. A. Burdick , “Computational Modeling and Experimental Characterization of Extrusion Printing Into Suspension Baths,” Advanced Healthcare Materials 11, no. 7 (2021): 2101679, 10.1002/adhm.202101679. PMC8986563 34699689

[42] N. S. Kajave , T. Schmitt , T. U. Nguyen , A. K. Gaharwar , and V. Kishore , “Bioglass Incorporated Methacrylated Collagen Bioactive Ink for 3D Printing of Bone Tissue,” Biomedical Materials 16, no. 3 (2021): 035003, 10.1088/1748-605X/abc744. PMC8326306 33142268

[43] A. M. Compaan , K. Song , W. Chai , and Y. Huang , “Cross‐Linkable Microgel Composite Matrix Bath for Embedded Bioprinting of Perfusable Tissue Constructs and Sculpting of Solid Objects,” ACS Applied Materials & Interfaces 12, no. 7 (2020): 7855–7868, 10.1021/acsami.9b15451. 31948226

[44] A. K. Grosskopf , R. L. Truby , H. Kim , A. Perazzo , J. A. Lewis , and H. A. Stone , “Viscoplastic Matrix Materials for Embedded 3D Printing,” ACS Applied Materials & Interfaces 10, no. 27 (2018): 23353–23361, 10.1021/acsami.7b19818. 29493215

[45] Y. Jin , W. Chai , and Y. Huang , “Printability Study of Hydrogel Solution Extrusion in Nanoclay Yield‐Stress Bath During Printing‐Then‐Gelation Biofabrication,” Materials Science and Engineering: C 80 (2017): 313–325, 10.1016/j.msec.2017.05.144. 28866170

[46] H. Li , Y. J. Tan , R. Kiran , S. B. Tor , and K. Zhou , “Submerged and Non‐Submerged 3D Bioprinting Approaches for the Fabrication of Complex Structures With the Hydrogel Pair GelMA and Alginate/Methylcellulose,” Additive Manufacturing 37 (2021): 101640, 10.1016/j.addma.2020.101640.

[47] C. Wang , J. R. Honiball , J. Lin , et al., “Infiltration From Suspension Systems Enables Effective Modulation of 3D Scaffold Properties in Suspension Bioprinting,” ACS Applied Materials & Interfaces 14, no. 24 (2022): 27575–27588, 10.1021/acsami.2c04163. 35674114

[48] Q. Li , Z. Jiang , L. Ma , et al., “A Versatile Embedding Medium for Freeform Bioprinting With Multi‐Crosslinking Methods,” Biofabrication 14, no. 3 (2022): 035022, 10.1088/1758-5090/ac7909. 35705061

[49] B. Wu , Z. Zeng , Y. Fang , et al., “Omnidirectional Elastic Constraint‐Based Embedded 3D Printing in Non‐Yield Stress Fluids for Engineering Complex Tissues,” Materials Today 80 (2024): 74–86, 10.1016/j.mattod.2024.08.005.

[50] X. Wei , Y. Wu , K. Chen , L. Wang , and M. Xu , “Embedded Bioprinted Multicellular Spheroids Modeling Pancreatic Cancer Bioarchitecture Towards Advanced Drug Therapy,” Journal of Materials Chemistry B 12, no. 7 (2024): 1788–1797, 10.1039/d3tb02913a. 38268422

[51] T. E. Greenwood , S. E. Hatch , M. B. Colton , and S. L. Thomson , “3D Printing Low‐Stiffness Silicone Within a Curable Support Matrix,” Additive Manufacturing 37 (2021): 101681, 10.1016/j.addma.2020.101681. 33718006 PMC7946128

[52] W. Hua , K. Mitchell , L. S. Kariyawasam , et al., “Three‐Dimensional Printing in Stimuli‐Responsive Yield‐Stress Fluid With an Interactive Dual Microstructure,” ACS Applied Materials & Interfaces 14, no. 34 (2022): 39420–39431, 10.1021/acsami.2c12465. 35973232

[53] E. Pairam , H. Le , and A. Fernandez‐Nieves , “Stability of Toroidal Droplets Inside Yield Stress Materials,” Physical Review E 90, no. 2 (2014): 021002, 10.1103/PhysRevE.90.021002. 25215681

[54] A. M'Barki , L. Bocquet , and A. Stevenson , “Linking Rheology and Printability for Dense and Strong Ceramics by Direct Ink Writing,” Scientific Reports 7, no. 1 (2017): 6017, 10.1038/s41598-017-06115-0. 28729671 PMC5519596

[55] G. Luo , Y. Yu , Y. Yuan , X. Chen , Z. Liu , and T. Kong , “Freeform, Reconfigurable Embedded Printing of All‐Aqueous 3D Architectures,” Advanced Materials 31, no. 49 (2019): 1904631, 10.1002/adma.201904631. 31609497

[56] M. Mahmoudi , S. R. Burlison , S. Moreno , and M. Minary‐Jolandan , “Additive‐Free and Support‐Free 3D Printing of Thermosetting Polymers With Isotropic Mechanical Properties,” ACS Applied Materials & Interfaces 13, no. 4 (2021): 5529–5538, 10.1021/acsami.0c19608. 33476138

[57] S. Shin and J. Hyun , “Rheological Properties of Cellulose Nanofiber Hydrogel for High‐Fidelity 3D Printing,” Carbohydrate Polymers 263 (2021): 117976, 10.1016/j.carbpol.2021.117976. 33858573

[58] B. Zhang , Q. Xue , H. Y. Hu , et al., “Integrated 3D Bioprinting‐Based Geometry‐Control Strategy for Fabricating Corneal Substitutes,” Journal of Zhejiang University‐Science B 20, no. 12 (2019): 945–959, 10.1631/jzus.B1900190. 31749342 PMC6885410

[59] M. B. Riffe , M. D. Davidson , G. Seymour , et al., “Multi‐Material Volumetric Additive Manufacturing of Hydrogels Using Gelatin as a Sacrificial Network and 3D Suspension Bath,” Advanced Materials 36, no. 34 (2024): 2309026, 10.1002/adma.202309026. PMC11259577 38243918

[60] D. Ribezzi , M. Gueye , S. Florczak , et al., “Shaping Synthetic Multicellular and Complex Multimaterial Tissues via Embedded Extrusion‐Volumetric Printing of Microgels,” Advanced Materials 35, no. 36 (2023): 2301673, 10.1002/adma.202301673. 37269532

[61] J. Lewicki , J. Bergman , C. Kerins , and O. Hermanson , “Optimization of 3D Bioprinting of Human Neuroblastoma Cells Using Sodium Alginate Hydrogel,” Bioprinting 16 (2019): e00053, 10.1016/j.bprint.2019.e00053.

[62] C. Bilici , A. G. Tatar , E. Senturk , C. Dikyol , and B. Koc , “Bisulfite‐Initiated Crosslinking of Gelatin Methacryloyl Hydrogels for Embedded 3D Bioprinting,” Biofabrication 14, no. 2 (2022): 025011, 10.1088/1758-5090/ac4dd9. 35062010

[63] R. Karyappa and M. Hashimoto , “Freeform Polymer Precipitation in Microparticulate Gels,” ACS Applied Polymer Materials 3, no. 2 (2021): 908–919, 10.1021/acsapm.0c01208.

[64] L. Hao , X. Tao , M. Feng , et al., “Stepwise Multi‐Cross‐Linking Bioink for 3D Embedded Bioprinting to Promote Full‐Thickness Wound Healing,” ACS Applied Materials & Interfaces 15, no. 20 (2023): 24034–24046, 10.1021/acsami.3c00688. 37159919

[65] Y. Jin , A. Compaan , T. Bhattacharjee , and Y. Huang , “Granular Gel Support‐Enabled Extrusion of Three‐Dimensional Alginate and Cellular Structures,” Biofabrication 8, no. 2 (2016): 025016, 10.1088/1758-5090/8/2/025016. 27257095

[66] H. Savoji , L. Davenport Huyer , M. H. Mohammadi , et al., “3D Printing of Vascular Tubes Using Bioelastomer Prepolymers by Freeform Reversible Embedding,” ACS Biomaterials Science & Engineering 6, no. 3 (2020): 1333–1343, 10.1021/acsbiomaterials.9b00676. 33455372

[67] S. Lee , E. S. Sani , A. R. Spencer , Y. Guan , A. S. Weiss , and N. Annabi , “Human‐Recombinant‐Elastin‐Based Bioinks for 3D Bioprinting of Vascularized Soft Tissues,” Advanced Materials 32, no. 45 (2020): 2003915, 10.1002/adma.202003915. PMC7658039 33000880

[68] S. Shin , H. Kwak , D. Shin , and J. Hyun , “Solid Matrix‐Assisted Printing for Three‐Dimensional Structuring of a Viscoelastic Medium Surface,” Nature Communications 10, no. 1 (2019): 4650, 10.1038/s41467-019-12585-9. PMC6789121 31604956

[69] J. Kim and J. Hyun , “Soft Magnetostrictive Actuator String With Cellulose Nanofiber Skin,” ACS Applied Materials & Interfaces 13, no. 37 (2021): 43904–43913, 10.1021/acsami.1c09410. 34495638

[70] T. Bhattacharjee , C. P. Kabb , C. S. O'Bryan , et al., “Polyelectrolyte Scaling Laws for Microgel Yielding Near Jamming,” Soft Matter 14, no. 9 (2018): 1559–1570, 10.1039/c7sm01518f. 29450413

[71] C. D. Morley , S. T. Ellison , T. Bhattacharjee , et al., “Quantitative Characterization of 3D Bioprinted Structural Elements Under Cell Generated Forces,” Nature Communications 10, no. 1 (2019): 3029, 10.1038/s41467-019-10919-1. PMC6620298 31292444

[72] J. Zeng , Z. Xie , Y. Dekishima , S. Kuwagaki , N. Sakai , and M. Matsusaki , ““Out‐of‐the‐Box” Granular Gel Bath Based on Cationic Polyvinyl Alcohol Microgels for Embedded Extrusion Printing,” Macromolecular Rapid Communications 44, no. 8 (2023): 2300025, 10.1002/marc.202300025. 36794543

[73] O. Jeon , Y. Bin Lee , T. J. Hinton , A. W. Feinberg , and E. Alsberg , “Cryopreserved Cell‐Laden Alginate Microgel Bioink for 3D Bioprinting of Living Tissues,” Materials Today Chemistry 12 (2019): 61–70, 10.1016/j.mtchem.2018.11.009. 30778400 PMC6377241

[74] J. Zhu , Y. He , L. Kong , et al., “Digital Assembly of Spherical Viscoelastic Bio‐Ink Particles,” Advanced Functional Materials 32, no. 6 (2021): 2109004, 10.1002/adfm.202109004.

[75] E. Mirdamadi , J. W. Tashman , D. J. Shiwarski , R. N. Palchesko , and A. W. Feinberg , “FRESH 3D Bioprinting a Full‐Size Model of the Human Heart,” ACS Biomaterials Science & Engineering 6, no. 11 (2020): 6453–6459, 10.1021/acsbiomaterials.0c01133. 33449644

[76] W. Sun , J. W. Tashman , D. J. Shiwarski , A. W. Feinberg , and V. A. Webster‐Wood , “Long‐Fiber Embedded Hydrogel 3D Printing for Structural Reinforcement,” ACS Biomaterials Science & Engineering 8, no. 1 (2022): 303–313, 10.1021/acsbiomaterials.1c00908. 34860495 PMC9206824

[77] S. M. Hull , C. D. Lindsay , L. G. Brunel , et al., “3D Bioprinting Using UNIversal Orthogonal Network (UNION) Bioinks,” Advanced Functional Materials 31, no. 7 (2021): 2007983, 10.1002/adfm.202007983. 33613150 PMC7888563

[78] Y. Li , S. Cheng , H. Shi , et al., “3D Embedded Bioprinting of Large‐Scale Intestine With Complex Structural Organization and Blood Capillaries,” Biofabrication 16, no. 4 (2024): 045001, 10.1088/1758-5090/ad5b1b. 38914075

[79] E. Mirdamadi , N. Muselimyan , P. Koti , H. Asfour , and N. Sarvazyan , “Agarose Slurry as a Support Medium for Bioprinting and Culturing Freestanding Cell‐Laden Hydrogel Constructs,” 3D Printing and Additive Manufacturing 6, no. 3 (2019): 158–164, 10.1089/3dp.2018.0175. 31860224 PMC6594419

[80] B. B. Mendes , M. Gomez‐Florit , A. G. Hamilton , et al., “Human Platelet Lysate‐Based Nanocomposite Bioink for Bioprinting Hierarchical Fibrillar Structures,” Biofabrication 12, no. 1 (2019): 015012, 10.1088/1758-5090/ab33e8. 31323659

[81] M. E. Prendergast , S. J. Heo , R. L. Mauck , and J. A. Burdick , “Suspension Bath Bioprinting and Maturation of Anisotropic Meniscal Constructs,” Biofabrication 15, no. 3 (2023): 035003, 10.1088/1758-5090/acc3c3. PMC10156462 36913724

[82] M. E. Prendergast , M. D. Davidson , and J. A. Burdick , “A Biofabrication Method to Align Cells Within Bioprinted Photocrosslinkable and Cell‐Degradable Hydrogel Constructs via Embedded Fibers,” Biofabrication 13, no. 4 (2021): 044108, 10.1088/1758-5090/ac25cc. PMC8603602 34507304

[83] Z. T. Xie , J. Zeng , D. H. Kang , et al., “3D Printing of Collagen Scaffold With Enhanced Resolution in a Citrate‐Modulated Gellan Gum Microgel Bath,” Advanced Healthcare Materials 12, no. 27 (2023): 2301090, 10.1002/adhm.202301090. 37143444

[84] S. R. Moxon , M. E. Cooke , S. C. Cox , et al., “Suspended Manufacture of Biological Structures,” Advanced Materials 29, no. 13 (2017): 1605594, 10.1002/adma.201605594. 28145596

[85] G. Cidonio , M. Cooke , M. Glinka , J. I. Dawson , L. Grover , and R. O. C. Oreffo , “Printing Bone in a Gel: Using Nanocomposite Bioink to Print Functionalised Bone Scaffolds,” Materials Today Bio 4 (2019): 100028, 10.1016/j.mtbio.2019.100028. PMC6894340 31853520

[86] J. J. Senior , M. E. Cooke , L. M. Grover , and A. M. Smith , “Fabrication of Complex Hydrogel Structures Using Suspended Layer Additive Manufacturing (SLAM),” Advanced Functional Materials 29, no. 49 (2019): 1904845, 10.1002/adfm.201904845.

[87] J. Zeng , N. Kasahara , Z. Xie , et al., “Comparative Analysis of the Residues of Granular Support Bath Materials on Printed Structures in Embedded Extrusion Printing,” Biofabrication 15, no. 3 (2023): 035013, 10.1088/1758-5090/acce09. 37072004

[88] S. Chen , Q. Shi , T. Jang , et al., “Engineering Natural Pollen Grains as Multifunctional 3D Printing Materials,” Advanced Functional Materials 31, no. 49 (2021): 2106276, 10.1002/adfm.202106276.

[89] W. Hua , C. Zhang , L. Raymond , et al., “3D Printing‐Based Full‐Scale Human Brain for Diverse Applications,” Brain‐X 1, no. 1 (2023): 5, 10.1002/brx2.5. PMC10564551 37818250

[90] A. Basu , A. Saha , C. Goodman , R. T. Shafranek , and A. Nelson , “Catalytically Initiated Gel‐in‐Gel Printing of Composite Hydrogels,” ACS Applied Materials & Interfaces 9, no. 46 (2017): 40898–40904, 10.1021/acsami.7b14177. 29091399

[91] A. Colly , C. Marquette , and E. J. Courtial , “Poloxamer/Poly(Ethylene Glycol) Self‐Healing Hydrogel for High‐Precision Freeform Reversible Embedding of Suspended Hydrogel,” Langmuir 37, no. 14 (2021): 4154–4162, 10.1021/acs.langmuir.1c00018. 33787263

[92] F. Afghah , M. Altunbek , C. Dikyol , and B. Koc , “Preparation and Characterization of Nanoclay‐Hydrogel Composite Support‐Bath for Bioprinting of Complex Structures,” Scientific Reports 10, no. 1 (2020): 5257, 10.1038/s41598-020-61606-x. 32210259 PMC7093553

[93] L. Raymond , W. Hua , N. Valentin , et al., “Coaxial Nozzle‐Assisted Embedded 3D Printing of Single‐Layered Channels Within a Yield‐Stress Matrix Bath,” Journal of Manufacturing Science and Engineering 146, no. 1 (2024): 011010, 10.1115/1.4063452.

[94] H. Ding and R. Chang , “Printability Study of Bioprinted Tubular Structures Using Liquid Hydrogel Precursors in a Support Bath,” Applied Sciences 8, no. 3 (2018): 403, 10.3390/app8030403.

[95] M. J. Rodriguez , T. A. Dixon , E. Cohen , W. Huang , F. G. Omenetto , and D. L. Kaplan , “3D Freeform Printing of Silk Fibroin,” Acta Biomaterialia 71 (2018): 379–387, 10.1016/j.actbio.2018.02.035. 29550442 PMC5899947

[96] F. Cheng , X. Cao , H. Li , et al., “Generation of Cost‐Effective Paper‐Based Tissue Models Through Matrix‐Assisted Sacrificial 3D Printing,” Nano Letters 19, no. 6 (2019): 3603–3611, 10.1021/acs.nanolett.9b00583. 31010289 PMC6820351

[97] S. M. Bakht , M. Gomez‐Florit , T. Lamers , R. L. Reis , R. M. A. Domingues , and M. E. Gomes , “3D Bioprinting of Miniaturized Tissues Embedded in Self‐Assembled Nanoparticle‐Based Fibrillar Platforms,” Advanced Functional Materials 31, no. 46 (2021): 2104245, 10.1002/adfm.202104245.

[98] H. Zhou , P. Liu , Z. Gao , et al., “Simultaneous Multimaterial Multimethod Bioprinting,” Bio‐Design and Manufacturing 5, no. 3 (2022): 433–436, 10.1007/s42242-022-00203-5.

[99] Z. Gao , J. Yin , P. Liu , et al., “Simultaneous Multi‐Material Embedded Printing for 3D Heterogeneous Structures,” International Journal of Extreme Manufacturing 5, no. 3 (2023): 035001, 10.1088/2631-7990/acd285.

[100] Z. Jiang , B. Jin , Z. Liang , et al., “Liver Bioprinting Within a Novel Support Medium With Functionalized Spheroids, Hepatic Vein Structures, and Enhanced Post‐Transplantation Vascularization,” Biomaterials 311 (2024): 122681, 10.1016/j.biomaterials.2024.122681. 38944968

[101] L. Lian , C. Zhou , G. Tang , et al., “Uniaxial and Coaxial Vertical Embedded Extrusion Bioprinting,” Advanced Healthcare Materials 11, no. 9 (2021): 2102411, 10.1002/adhm.202102411. 34860472

[102] Y. C. Li , Y. A. Jodat , R. Samanipour , et al., “Toward a Neurospheroid Niche Model: Optimizing Embedded 3D Bioprinting for Fabrication of Neurospheroid Brain‐Like Co‐Culture Constructs,” Biofabrication 13 (2020): 015014, 10.1088/1758-5090/abc1be. PMC8387028 33059333

[103] M. Ye , B. Lu , X. Zhang , B. Li , Z. Xiong , and T. Zhang , “Coaxial Embedded Printing of Gelatin Methacryloyl–Alginate Double Network Hydrogel for Multilayer Vascular Tubes,” Chinese Journal of Mechanical Engineering: Additive Manufacturing Frontiers 1, no. 2 (2022): 100024, 10.1016/j.cjmeam.2022.100024.

[104] C. B. Highley , K. H. Song , A. C. Daly , and J. A. Burdick , “Jammed Microgel Inks for 3D Printing Applications,” Advanced Science 6, no. 1 (2019): 1801076, 10.1002/advs.201801076. 30643716 PMC6325587

[105] L. Shi , H. Carstensen , K. Hölzl , et al., “Dynamic Coordination Chemistry Enables Free Directional Printing of Biopolymer Hydrogel,” Chemistry of Materials 29, no. 14 (2017): 5816–5823, 10.1021/acs.chemmater.7b00128.

[106] G. Gao , H. Kim , B. S. Kim , et al., “Tissue‐Engineering of Vascular Grafts Containing Endothelium and Smooth‐Muscle Using Triple‐Coaxial Cell Printing,” Applied Physics Reviews 6, no. 4 (2019): 041402, 10.1063/1.5099306.

[107] G. Gao , J. H. Lee , J. Jang , et al., “Tissue Engineered Bio‐Blood‐Vessels Constructed Using a Tissue‐Specific Bioink and 3D Coaxial Cell Printing Technique: A Novel Therapy for Ischemic Disease,” Advanced Functional Materials 27, no. 33 (2017): 1700798, 10.1002/adfm.201700798.

[108] G. Gao , J. Y. Park , B. S. Kim , J. Jang , and D. W. Cho , “Coaxial Cell Printing of Freestanding, Perfusable, and Functional In Vitro Vascular Models for Recapitulation of Native Vascular Endothelium Pathophysiology,” Advanced Healthcare Materials 7, no. 23 (2018): 1801102, 10.1002/adhm.201801102. 30370670

[109] D. Kim , H. Hwangbo , and G. Kim , “Engineered Myoblast‐Laden Collagen Filaments Fabricated Using a Submerged Bioprinting Process to Obtain Efficient Myogenic Activities,” Biomacromolecules 22, no. 12 (2021): 5042–5051, 10.1021/acs.biomac.1c01006. 34783537

[110] A. E. Figueroa‐Milla , W. DeMaria , D. Wells , O. Jeon , E. Alsberg , and M. W. Rolle , “Vascular Tissues Bioprinted With Smooth Muscle Cell‐Only Bioinks in Support Baths Mimic Features of Native Coronary Arteries,” Biofabrication 16, no. 4 (2024): 045033, 10.1088/1758-5090/ad6d8f. 39121893

[111] L. Shao , J. Jiang , C. Yuan , X. Zhang , L. Gu , and X. Wang , “Omnidirectional Anisotropic Embedded 3D Bioprinting,” Materials Today Bio 27 (2024): 101160, 10.1016/j.mtbio.2024.101160. PMC11326905 39155942

[112] W. Shi , S. Mirza , M. Kuss , et al., “Embedded Bioprinting of Breast Tumor Cells and Organoids Using Low‐Concentration Collagen‐Based Bioinks,” Advanced Healthcare Materials 12, no. 26 (2023): 2300905, 10.1002/adhm.202300905. 37422447 PMC10592394

[113] D. F. Duarte Campos , A. Blaeser , M. Weber , et al., “Three‐Dimensional Printing of Stem Cell‐Laden Hydrogels Submerged in a Hydrophobic High‐Density Fluid,” Biofabrication 5, no. 1 (2013): 015003, 10.1088/1758-5082/5/1/015003. 23172592

[114] X. Deng , C. Qi , S. Meng , et al., “All‐Aqueous Embedded 3D Printing for Freeform Fabrication of Biomimetic 3D Constructs,” Advanced Materials 36, no. 50 (2024): 2406825, 10.1002/adma.202406825. 39520386

[115] J. Forth , X. Liu , J. Hasnain , et al., “Reconfigurable Printed Liquids,” Advanced Materials 30, no. 16 (2018): 1707603, 10.1002/adma.201707603. 29573293

[116] S. Zhang , C. Qi , W. Zhang , et al., “In Situ Endothelialization of Free‐Form 3D Network of Interconnected Tubular Channels via Interfacial Coacervation by Aqueous‐in‐Aqueous Embedded Bioprinting,” Advanced Materials 35, no. 7 (2023): 2209263, 10.1002/adma.202209263. 36448877

[117] G. Xie , J. Forth , Y. Chai , P. D. Ashby , B. A. Helms , and T. P. Russell , “Compartmentalized, All‐Aqueous Flow‐Through‐Coordinated Reaction Systems,” Chemistry 5, no. 10 (2019): 2678–2690, 10.1016/j.chempr.2019.07.016.

[118] C. Qi , T. Zhou , X. Wu , et al., “Micro‐Nano‐Fabrication of Green Functional Materials by Multiphase Microfluidics for Environmental and Energy Applications,” Green Energy & Environment 9, no. 8 (2024): 1199–1219, 10.1016/j.gee.2023.05.012.

[119] H. Wang , X. Zhou , J. Wang , X. Zhang , M. Zhu , and H. Wang , “Fabrication of Channeled Scaffolds Through Polyelectrolyte Complex (PEC) Printed Sacrificial Templates for Tissue Formation,” Bioactive Materials 17 (2022): 261–275, 10.1016/j.bioactmat.2022.01.030. 35386455 PMC8965085

[120] S. Emonds , J. Kamp , J. Borowec , H. Roth , and M. Wessling , “Polyelectrolyte Complex Tubular Membranes via a Salt Dilution Induced Phase Inversion Process,” Advanced Engineering Materials 23, no. 5 (2021): 2001401, 10.1002/adem.202001401.

[121] M. Khoonkari , J. E. Sayed , M. Oggioni , et al., “Bioinspired Processing: Complex Coacervates as Versatile Inks for 3D Bioprinting,” Advanced Materials 35, no. 28 (2023): 2210769, 10.1002/adma.202210769. 36916861

[122] D. Wang , S. Maharjan , X. Kuang , et al., “Microfluidic Bioprinting of Tough Hydrogel‐Based Vascular Conduits for Functional Blood Vessels,” Science Advances 8, no. 43 (2022): abq6900, 10.1126/sciadv.abq6900. PMC9604524 36288300

[123] C. Xu , W. Chai , Y. Huang , and R. R. Markwald , “Scaffold‐Free Inkjet Printing of Three‐Dimensional Zigzag Cellular Tubes,” Biotechnology and Bioengineering 109, no. 12 (2012): 3152–3160, 10.1002/bit.24591. 22767299

[124] C. Xu , K. Christensen , Z. Zhang , Y. Huang , J. Fu , and R. R. Markwald , “Predictive Compensation‐Enabled Horizontal Inkjet Printing of Alginate Tubular Constructs,” Manufacturing Letters 1, no. 1 (2013): 28–32, 10.1016/j.mfglet.2013.09.003.

[125] P. C. W. M. van der Valk and M. J. Mirzaali , “Non‐Planar Additive Manufacturing With Hydrogels: A Review of Flow Control and Toolpath Strategies,” npj Soft Matter 1, no. 1 (2025): 5, 10.1038/s44431-025-00006-5.

[126] X. Guidetti , E. C. Balta , Y. Nagel , H. Yin , A. Rupenyan , and J. Lygeros , “Stress Flow Guided Non‐Planar Print Trajectory Optimization for Additive Manufacturing of Anisotropic Polymers,” Additive Manufacturing 72 (2023): 103628, 10.1016/j.addma.2023.103628.

[127] M. S. Chaudhry and A. Czekanski , “Surface Slicing and Toolpath Planning for In‐Situ Bioprinting of Skin Implants,” Biofabrication 16, no. 2 (2024): 025030, 10.1088/1758-5090/ad30c4. 38447215

[128] Y. Shan , Y. Shui , J. Hua , and H. Mao , “Additive Manufacturing of Non‐Planar Layers Using Isothermal Surface Slicing,” Journal of Manufacturing Processes 86 (2023): 326–335, 10.1016/j.jmapro.2022.12.054.

[129] T. Zhang , G. Fang , Y. Huang , et al., “S^3^‐Slicer: A General Slicing Framework for Multi‐Axis 3D Printing,” ACM Transactions on Graphics 41, no. 6 (2022): 1–15, 10.1145/3550454.3555516.

[130] B. J. Albert , C. Wang , C. Williams , and J. T. Butcher , “Non‐Planar Embedded 3D Printing for Complex Hydrogel Manufacturing,” Bioprinting 28 (2022): e00242, 10.1016/j.bprint.2022.e00242.

[131] R. G. T. Romero , M. B. Colton , and S. L. Thomson , “3D‐Printed Synthetic Vocal Fold Models,” Journal of Voice 35, no. 5 (2021): 685–694, 10.1016/j.jvoice.2020.01.030. 32312610 PMC7643278

[132] G. Ruocco , E. Marcello , C. Paoletti , et al., “Fibrous Bioinks for Bioprinting Anisotropic Micro‐and Nanoscale Scaffolds: A Novel Strategy for In Vitro Skeletal Muscle Engineering,” International Journal of Bioprinting 12, no. 1 (2025): 323–347, 10.36922/ijb025440455.

[133] C. Radeke , R. Pons , M. Mihajlovic , et al., “Transparent and Cell‐Guiding Cellulose Nanofiber 3D Printing Bioinks,” ACS Applied Materials & Interfaces 15, no. 2 (2023): 2564–2577, 10.1021/acsami.2c16126. 36598781

[134] J. H. Ahrens , S. G. M. Uzel , M. Skylar‐Scott , et al., “Programming Cellular Alignment in Engineered Cardiac Tissue via Bioprinting Anisotropic Organ Building Blocks,” Advanced Materials 34, no. 26 (2022): 2200217, 10.1002/adma.202200217. 35451188

[135] S. Propst and J. Mueller , “Time Code for Multifunctional 3D Printhead Controls,” Nature Communications 16, no. 1 (2025): 1035, 10.1038/s41467-025-56140-1. PMC11763051 39863581

[136] E. Kucukdeger , Y. Zhang , J. Zhang , Y. Liu , X. Jia , and B. N. Johnson , “Automated Characterization of Macroscale Tissue 3D Spatial Material Properties via Robotically‐Directed Impedimetric Sensing,” IEEE Transactions on Automation Science and Engineering 21, no. 3 (2024): 3408–3420, 10.1109/tase.2023.3279338.

[137] L. Xiaorui , Z. Fuyin , W. Xudong , et al., “Biomaterial Inks for Extrusion‐Based 3D Bioprinting: Property, Classification, Modification, and Selection,” International Journal of Bioprinting 9, no. 2 (2023): 649, 10.18063/ijb.v9i2.649. 37065674 PMC10090818

[138] N. Paxton , W. Smolan , T. Bock , F. Melchels , J. Groll , and T. Jungst , “Proposal to Assess Printability of Bioinks for Extrusion‐Based Bioprinting and Evaluation of Rheological Properties Governing Bioprintability,” Biofabrication 9, no. 4 (2017): 044107, 10.1088/1758-5090/aa8dd8. 28930091

[139] J. Emmermacher , D. Spura , J. Cziommer , et al., “Engineering Considerations on Extrusion‐Based Bioprinting: Interactions of Material Behavior, Mechanical Forces and Cells in the Printing Needle,” Biofabrication 12, no. 2 (2020): 025022, 10.1088/1758-5090/ab7553. 32050179

[140] C. Yan , M. E. Mackay , K. Czymmek , R. P. Nagarkar , J. P. Schneider , and D. J. Pochan , “Injectable Solid Peptide Hydrogel as a Cell Carrier: Effects of Shear Flow on Hydrogels and Cell Payload,” Langmuir 28, no. 14 (2012): 6076–6087, 10.1021/la2041746. 22390812 PMC4196894

[141] N. Thattaruparambil Raveendran , C. Vaquette , C. Meinert , D. Samuel Ipe , and S. Ivanovski , “Optimization of 3D Bioprinting of Periodontal Ligament Cells,” Dental Materials 35, no. 12 (2019): 1683–1694, 10.1016/j.dental.2019.08.114. 31601443

[142] S. Han , C. M. Kim , S. Jin , and T. Y. Kim , “Study of the Process‐Induced Cell Damage in Forced Extrusion Bioprinting,” Biofabrication 13, no. 3 (2021): 035048, 10.1088/1758-5090/ac0415. 34020427

[143] R. Chang , J. Nam , and W. Sun , “Effects of Dispensing Pressure and Nozzle Diameter on Cell Survival From Solid Freeform Fabrication–Based Direct Cell Writing,” Tissue Engineering Part A 14, no. 1 (2008): 41–48, 10.1089/ten.a.2007.0004. 18333803

[144] L. Ouyang , R. Yao , Y. Zhao , and W. Sun , “Effect of Bioink Properties on Printability and Cell Viability for 3D Bioplotting of Embryonic Stem Cells,” Biofabrication 8, no. 3 (2016): 035020, 10.1088/1758-5090/8/3/035020. 27634915

[145] B. A. Aguado , W. Mulyasasmita , J. Su , K. J. Lampe , and S. C. Heilshorn , “Improving Viability of Stem Cells During Syringe Needle Flow Through the Design of Hydrogel Cell Carriers,” Tissue Engineering Part A 18, no. 7‐8 (2012): 806–815, 10.1089/ten.TEA.2011.0391. 22011213 PMC3313609

[146] L. Ning , N. Betancourt , D. J. Schreyer , and X. Chen , “Characterization of Cell Damage and Proliferative Ability During and After Bioprinting,” ACS Biomaterials Science & Engineering 4, no. 11 (2018): 3906–3918, 10.1021/acsbiomaterials.8b00714. 33429605

[147] T. Billiet , E. Gevaert , T. De Schryver , M. Cornelissen , and P. Dubruel , “The 3D Printing of Gelatin Methacrylamide Cell‐Laden Tissue‐Engineered Constructs With High Cell Viability,” Biomaterials 35, no. 1 (2014): 49–62, 10.1016/j.biomaterials.2013.09.078. 24112804

[148] M. Coutanceau and J.‐R. Defaye , “Circular Cylinder Wake Configurations: A Flow Visualization Survey,” Applied Mechanics Reviews 44, no. 6 (1991): 255–305, 10.1115/1.3119504.

[149] A. Thom , “The Flow Past Circular Cylinders at Low Speeds,” Proceedings of the Royal Society of London Series A, Containing Papers of a Mathematical and Physical Character 141, no. 845 (1933): 651–669, 10.1098/rspa.1933.0146.

[150] C. S. O'Bryan , T. Bhattacharjee , S. Hart , et al., “Self‐Assembled Micro‐Organogels for 3D Printing Silicone Structures,” Science Advances 3, no. 5 (2017): 1602800, 10.1126/sciadv.1602800. PMC5425239 28508071

[151] Y. Jin , A. Compaan , W. Chai , and Y. Huang , “Functional Nanoclay Suspension for Printing‐Then‐Solidification of Liquid Materials,” ACS Applied Materials & Interfaces 9, no. 23 (2017): 20057–20066, 10.1021/acsami.7b02398. 28534614

[152] O. Jeon , Y. B. Lee , H. Jeong , S. J. Lee , D. Wells , and E. Alsberg , “Individual Cell‐Only Bioink and Photocurable Supporting Medium for 3D Printing and Generation of Engineered Tissues With Complex Geometries,” Materials Horizons 6, no. 8 (2019): 1625–1631, 10.1039/c9mh00375d. 32864142 PMC7453938

[153] Y. Jin , K. Song , N. Gellermann , and Y. Huang , “Printing of Hydrophobic Materials in Fumed Silica Nanoparticle Suspension,” ACS Applied Materials & Interfaces 11, no. 32 (2019): 29207–29217, 10.1021/acsami.9b07433. 31333016

[154] C. S. O'Bryan , T. Bhattacharjee , S. R. Niemi , et al., “Three‐Dimensional Printing With Sacrificial Materials for Soft Matter Manufacturing,” MRS Bulletin 42, no. 08 (2017): 571–577, 10.1557/mrs.2017.167.

[155] Q. Wu , F. Zhu , Z. Wu , et al., “Suspension Printing of Liquid Metal in Yield‐Stress Fluid for Resilient 3D Constructs With Electromagnetic Functions,” npj Flexible Electronics 6, no. 1 (2022): 50, 10.1038/s41528-022-00184-6.

[156] C. Zhang , W. Hua , K. Mitchell , et al., “Multiscale Embedded Printing of Engineered Human Tissue and Organ Equivalents,” Proceedings of the National Academy of Sciences USA 121, no. 9 (2024): 2313464121, 10.1073/pnas.2313464121. PMC10907305 38346211

[157] T. Li , J. Hou , L. Wang , et al., “Bioprinted Anisotropic Scaffolds With Fast Stress Relaxation Bioink for Engineering 3D Skeletal Muscle and Repairing Volumetric Muscle Loss,” Acta Biomaterialia 156 (2023): 21–36, 10.1016/j.actbio.2022.08.037. 36002128

[158] M. A. Stang , A. Lee , J. M. Bliley , et al., “Engineering 3D Skeletal Muscle Tissue With Complex Multipennate Myofiber Architectures,” bioRxiv (2025): 631119, 10.1101/2025.01.06.631119.

[159] M. G. Kontakis , M. Moulin , B. Andersson , et al., “Trabecular‐Bone Mimicking Osteoconductive Collagen Scaffolds: An Optimized 3D Printing Approach Using Freeform Reversible Embedding of Suspended Hydrogels,” 3D Printing in Medicine 11, no. 1 (2025): 11, 10.1186/s41205-025-00255-0. 40064747 PMC11895158

[160] S. Moxon , Z. McMurran , M. Kibble , M. Domingos , J. Gough , and S. Richardson , “3D Bioprinting of an Intervertebral Disc Tissue Analogue With a Highly Aligned Annulus Fibrosus via Suspended Layer Additive Manufacture,” Biofabrication 17, no. 1 (2024): 015005, 10.1088/1758-5090/ad8379. PMC11499629 39366424

[161] A. Abaci and M. Guvendiren , “3D Bioprinting of Dense Cellular Structures Within Hydrogels With Spatially Controlled Heterogeneity,” Biofabrication 16, no. 3 (2024): 035027, 10.1088/1758-5090/ad52f1. 38821144

[162] S. Li , J. Li , J. Xu , et al., “Removal‐Free and Multicellular Suspension Bath‐Based 3D Bioprinting,” Advanced Materials 36, no. 48 (2024): 2406891, 10.1002/adma.202406891. 39394784

[163] R. J. A. Moakes , J. J. Senior , T. E. Robinson , et al., “A Suspended Layer Additive Manufacturing Approach to the Bioprinting of Tri‐Layered Skin Equivalents,” APL Bioengineering 5, no. 4 (2021): 046103, 10.1063/5.0061361. 34888433 PMC8635740

[164] Y. Fang , M. Ji , Y. Yang , et al., “3D printing of Vascularized Hepatic Tissues With a High Cell Density and Heterogeneous Microenvironment,” Biofabrication 15, no. 4 (2023): 045004, 10.1088/1758-5090/ace5e0. 37429291

[165] N. K. Singh , J. Y. Kim , J. Jang , Y. K. Kim , and D. W. Cho , “3D Cell Printing of Advanced Vascularized Proximal Tubule‐on‐a‐Chip for Drug Induced Nephrotoxicity Advancement,” ACS Applied Bio Materials 6, no. 9 (2023): 3750–3758, 10.1021/acsabm.3c00421. 37606916

[166] S. Mehrotra , B. A. G. de Melo , M. Hirano , et al., “Nonmulberry Silk Based Ink for Fabricating Mechanically Robust Cardiac Patches and Endothelialized Myocardium‐on‐a‐Chip Application,” Advanced Functional Materials 30, no. 12 (2020): 1907436, 10.1002/adfm.201907436. 33071707 PMC7566555

[167] J. R. Sayers and P. R. Riley , “Heart Regeneration: Beyond New Muscle and Vessels,” Cardiovascular Research 117, no. 3 (2021): 727–742, 10.1093/cvr/cvaa320. 33241843

[168] J. C. Walcott and M. E. Davis , “Bioprinting Organoids for Functional Cardiac Constructs: Progress and Unmet Challenges,” International Journal of Bioprinting 11, no. 3 (2025): 85–114, 10.36922/ijb025150134.

[169] T. U. Esser , A. Anspach , K. A. Muenzebrock , et al., “Direct 3D‐Bioprinting of hiPSC‐Derived Cardiomyocytes to Generate Functional Cardiac Tissues,” Advanced Materials 35, no. 52 (2023): 2305911, 10.1002/adma.202305911. 37655652

[170] S. Finkel , S. Sweet , T. Locke , et al., “FRESH™ 3D Bioprinted Cardiac Tissue, a Bioengineered Platform for In Vitro Pharmacology,” APL Bioengineering 7, no. 4 (2023): 046113, 10.1063/5.0163363. 38046544 PMC10693443

[171] Y. Fang , B. Lu , R. Sun , et al., “Scalable Fabrication of Aligned Myocardial Tissues With Native‐Like Helical Architecture for Heart Repair,” Cell Biomaterials 2, no. 6 (2026): 100306, 10.1016/j.celbio.2025.100306.

[172] T. Fan , M. Jia , H. Liu , et al., “Engineering Strategies for the Construction of Oriented and Functional Skeletal Muscle Tissues,” Biofabrication 17, no. 2 (2025): 022013, 10.1088/1758-5090/adbfc2. 40073456

[173] J. Jiang , C. Yuan , X. Zhang , et al., “3D Bioprinting of Liquid High‐Cell‐Proportion Bioinks in Liquid Granular Bath,” Advanced Materials 36, no. 49 (2024): 2412127, 10.1002/adma.202412127. 39385640

[174] M. Jia , T. Fan , T. Jia , X. Liu , H. Liu , and Q. Gu , “Temporal and Spatial Regulation of Biomimetic Vascularization in 3D‐Printed Skeletal Muscles,” Bio‐Design and Manufacturing 7, no. 5 (2024): 597–610, 10.1007/s42242-024-00315-0.

[175] A. R. Hudson , D. J. Shiwarski , A. J. Kramer , and A. W. Feinberg , “Enhancing Viability in Static and Perfused 3D Tissue Constructs Using Sacrificial Gelatin Microparticles,” ACS Biomaterials Science & Engineering 11, no. 5 (2025): 2888–2897, 10.1021/acsbiomaterials.4c02169. 40194916 PMC12076283

[176] Y. J. Choi , Y. J. Jun , D. Y. Kim , et al., “A 3D Cell Printed Muscle Construct With Tissue‐Derived Bioink for the Treatment of Volumetric Muscle Loss,” Biomaterials 206 (2019): 160–169, 10.1016/j.biomaterials.2019.03.036. 30939408

[177] C. Di Bella , A. Fosang , D. M. Donati , G. G. Wallace , and P. F. Choong , “3D Bioprinting of Cartilage for Orthopedic Surgeons: Reading Between the Lines,” Frontiers Surgery 2 (2015): 39, 10.3389/fsurg.2015.00039. PMC4534805 26322314

[178] N. Raja , H. Park , C. W. Gal , A. Sung , Y. J. Choi , and H. S. Yun , “Support‐Less Ceramic 3D Printing of Bioceramic Structures Using a Hydrogel Bath,” Biofabrication 15, no. 3 (2023): 035006, 10.1088/1758-5090/acc903. 36996843

[179] S. S. Utami , N. Raja , J. Kim , et al., “Support‐Less 3D Bioceramic/Extracellular Matrix Printing in Sanitizer‐Based Hydrogel for Bone Tissue Engineering,” Biofabrication 17, no. 2 (2025): 025017, 10.1088/1758-5090/adb4a3. 39933193

[180] S. Romanazzo , T. G. Molley , S. Nemec , et al., “Synthetic Bone‐Like Structures Through Omnidirectional Ceramic Bioprinting in Cell Suspensions,” Advanced Functional Materials 31, no. 13 (2021): 2008216, 10.1002/adfm.202008216.

[181] B. Ayan , N. Celik , Z. Zhang , et al., “Aspiration‐Assisted Freeform Bioprinting of Pre‐Fabricated Tissue Spheroids in a Yield‐Stress Gel,” Communications Physics 3 (2020): 183, 10.1038/s42005-020-00449-4. 33251340 PMC7695349

[182] Y. Wu , X. Yang , T. Yuan , et al., “Development of Embedded Bioprinting for Fabricating Zonally Stratified Articular Cartilage,” International Journal of Bioprinting 10, no. 4 (2024): 3520, 10.36922/ijb.3520.

[183] G. K. Jalandhra , T. G. Molley , T. T. Hung , I. Roohani , and K. A. Kilian , “In Situ Formation of Osteochondral Interfaces Through “Bone‐Ink” Printing in Tailored Microgel Suspensions,” Acta Biomaterialia 156 (2023): 75–87, 10.1016/j.actbio.2022.08.052. 36055612

[184] M. J. Kibble , M. J. S. Ferreira , Y. H. Usta , et al., “Suspension Bioprinted Whole Intervertebral Disc Analogues Enable Regional Stiffness‐ and Hypoxia‐Regulated Matrix Secretion by Primary Human Nucleus Pulposus and Annulus Fibrosus Cells,” Acta Biomaterialia 200 (2025): 378–389, 10.1016/j.actbio.2025.05.015. 40339969

[185] D. M. Solis and A. Czekanski , “3D and 4D Additive Manufacturing Techniques for Vascular‐Like Structures – A Review,” Bioprinting 25 (2022): e00182, 10.1016/j.bprint.2021.e00182.

[186] A. Z. Nelson , B. Kundukad , W. K. Wong , S. A. Khan , and P. S. Doyle , “Embedded Droplet Printing in Yield‐Stress Fluids,” Proceedings of the National Academy of Sciences 117, no. 11 (2020): 5671–5679, 10.1073/pnas.1919363117. PMC7084155 32127482

[187] I. W. Zhang , L. S. Choi , N. E. Friend , et al., “Clickable PEG‐Norbornene Microgels Support Suspension Bioprinting and Microvascular Assembly,” Acta Biomaterialia 201 (2025): 283–296, 10.1016/j.actbio.2025.06.006. 40514334 PMC12277018

[188] K. J. Wolf , R. C. van Gaal , S. G. M. Uzel , et al., “Perfusable 3D Models of Ureteric Bud and Collecting Duct Tubules,” Cell Biomaterials 2, no. 3 (2026): 100297, 10.1016/j.celbio.2025.100297.

[189] I. Holland , J. Logan , J. Shi , C. McCormick , D. Liu , and W. Shu , “3D Biofabrication for Tubular Tissue Engineering,” Bio‐Design and Manufacturing 1, no. 2 (2018): 89–100, 10.1007/s42242-018-0013-2. 30546921 PMC6267270

[190] L. G. Brunel , F. Christakopoulos , D. Kilian , et al., “Embedded 3D Bioprinting of Collagen Inks Into Microgel Baths to Control Hydrogel Microstructure and Cell Spreading,” Advanced Healthcare Materials 13, no. 25 (2024): 2303325, 10.1002/adhm.202303325. PMC11192865 38134346

[191] P. Chaurasia , R. Singh , R. R. Kaushik , N. Yadav , and S. K. Mahto , “Fabrication and In Vivo Characterization of FRESH‐Based 3D Printed Chitosan Construct for Small Intestine Regeneration,” Biomedical Materials 20, no. 4 (2025): 045027, 10.1088/1748-605X/ade5e1. 40532727

[192] H. Zhang , Y. Luo , Z. Hu , et al., “Cation‐Crosslinked κ‐Carrageenan Sub‐Microgel Medium for High‐Quality Embedded Bioprinting,” Biofabrication 16, no. 2 (2024): 025009, 10.1088/1758-5090/ad1cf3. 38198708

[193] A. C. Daly , M. D. Davidson , and J. A. Burdick , “3D Bioprinting of High Cell‐Density Heterogeneous Tissue Models Through Spheroid Fusion Within Self‐Healing Hydrogels,” Nature Communications 12, no. 1 (2021): 753, 10.1038/s41467-021-21029-2. PMC7854667 33531489

[194] D. B. Kolesky , R. L. Truby , A. S. Gladman , T. A. Busbee , K. A. Homan , and J. A. Lewis , “3D Bioprinting of Vascularized, Heterogeneous Cell‐Laden Tissue Constructs,” Advanced Materials 26, no. 19 (2014): 3124–3130, 10.1002/adma.201305506. 24550124

[195] D. Youssef , S. Hassab‐Elnaby , and H. El‐Ghandoor , “Nanoscale Quantitative Surface Roughness Measurement of Articular Cartilage Using Second‐Order Statistical‐Based Biospeckle,” PLoS One 16, no. 1 (2021): 0246395, 10.1371/journal.pone.0246395. PMC7845957 33513197

[196] Z. Zhang , Z. Chen , Z. Li , et al., “Estimation of Skin Surface Roughness In Vivo Based on Optical Coherence Tomography Combined With Convolutional Neural Network,” Frontiers in Medicine 11 (2024): 1453405, 10.3389/fmed.2024.1453405. 39464272 PMC11502342

[197] D. G. Owen , T. Schenkel , D. E. T. Shepherd , and D. M. Espino , “Assessment of Surface Roughness and Blood Rheology on Local Coronary Haemodynamics: A Multi‐Scale Computational Fluid Dynamics Study,” Journal of the Royal Society Interface 17, no. 169 (2020): 20200327, 10.1098/rsif.2020.0327. 32781935 PMC7482556

[198] P. R. Bergstresser , R. J. Pariser , and J. R. Taylor , “Counting and Sizing of Epidermal Cells in Normal Human Skin,” Journal of Investigative Dermatology 70, no. 5 (1978): 280–284, 10.1111/1523-1747.ep12541516. 641379

[199] D. B. Cines , E. S. Pollak , C. A. Buck , et al., Blood, the Journal of the American Society of Hematology 91, no. 10 (1998): 3527, 10.1182/blood.V91.10.3527.

[200] Z. E. Wilson , A. Rostami‐Hodjegan , J. L. Burn , et al., “Inter‐Individual Variability in Levels of Human Microsomal Protein and Hepatocellularity per Gram of Liver,” British Journal of Clinical Pharmacology 56, no. 4 (2003): 433–440, 10.1046/j.1365-2125.2003.01881.x. 12968989 PMC1884378

[201] H. Naderi‐Meshkin , V. A. Cornelius , M. Eleftheriadou , K. N. Potel , W. A. W. Setyaningsih , and A. Margariti , “Vascular Organoids: Unveiling Advantages, Applications, Challenges, and Disease Modelling Strategies,” Stem Cell Research & Therapy 14, no. 1 (2023): 292, 10.1186/s13287-023-03521-2. 37817281 PMC10566155

[202] M. Wang , W. Li , J. Hao , et al., “Biomaterial‐Minimalistic Photoactivated Bioprinting of Cell‐Dense Tissues,” Cell 189, no. 1 (2026): 106–122.e26, 10.1016/j.cell.2025.11.012. 41365296 PMC13384749

[203] M. St Clair‐Glover , Z. Yue , and M. Dottori , “3D Printing for Neural Repair: Bridging the Gap in Regenerative Medicine,” Advanced Materials 37, no. 36 (2025): 07590, 10.1002/adma.202507590. PMC12422090 40736088

